# Impact of Vaccination and Public Health Measures on the Severity of SARS-CoV-2 Omicron Infections in China: A Systematic Review and Meta-Regression Analysis

**DOI:** 10.3390/vaccines13070747

**Published:** 2025-07-12

**Authors:** Can Wang, Liping Peng, Xiaotong Huang, Tim K. Tsang

**Affiliations:** WHO Collaborating Centre for Infectious Disease Epidemiology and Control, School of Public Health, Li Ka Shing Faculty of Medicine, The University of Hong Kong, Hong Kong SAR, China

**Keywords:** COVID-19, SARS-CoV-2, severity, Omicron, vaccine effectiveness, inactivated vaccines, public health measures

## Abstract

**Background:** Starting in early 2022, SARS-CoV-2 Omicron has driven large outbreaks in China, a predominantly infection-naive population with high inactivated vaccine coverage. This unique context provided a substantially less-confounded opportunity to evaluate how vaccination, public health, and social measures influenced severity. **Methods:** We systematically reviewed 86 studies (224 severity estimates) published from 2022 to 2024, reporting symptom and clinical severity outcomes (fever, cough, and sore throat; symptomatic, severe/critical, and fatal illness) of Omicron infections in China. Using meta-regression, we evaluated the associations of study setting, age group, vaccination status, predominant subvariants, and Oxford COVID-19 Government Response Tracker (OxCGRT) indices, including the Government Response Index (GRI), Containment and Health Index (CHI), and the Stringency Index (SI), with infection outcomes, adjusting for key confounders. **Results:** We found the primary or booster series of inactivated vaccines conferred strong protection against severe/critical illness (pooled relative risk (RR) 0.17 [95% CI: 0.09–0.33]) but did not reduce symptom frequency (RR 0.99 [95% CI: 0.95–1.02]). Each 10-unit increase in GRI or CHI was associated with 7% (95% CI: 1–12%) and 6% (95% CI: 1–10%) lower odds of symptomatic infection and 3% (95% CI: 1–4%) lower odds of severe/critical illness. Later subvariants (BA.5, BF.7, and XBB) showed 24–38% higher odds of upper respiratory symptoms versus BA.1. **Conclusions**: The data collection context significantly impacted severity estimates, with higher estimates from emergency hospitals. Overall, inactivated vaccines provided strong protection against severe/critical outcomes while stringent public health measures were associated with lower severity. Our findings underscore the importance of consistent and standardized protocols to produce reliable estimates of SARS-CoV-2 severity in evolving epidemiological contexts.

## 1. Introduction

Following the first locally transmitted Omicron case (BA.1) in Tianjin in January 2022, Severe Acute Respiratory Syndrome Coronavirus-2 (SARS-CoV-2) has driven multiple large epidemics across mainland China under a dynamic zero-COVID policy characterized by strict lockdowns and mass testing [1,2,3]. Prior to Omicron’s emergence, China had effectively contained regional outbreaks with documented minimal community transmission. This created a distinctive epidemiological setting: a population with an extremely low prevalence of prior infection and high rates of vaccine-induced immunity [4]. While some undetected infections were possible, China’s intensive testing and containment policies minimized their consequent hybrid immunity. Therefore, this unique context also provided a substantially less confounded opportunity to evaluate the intrinsic effectiveness of inactivated vaccines against Omicron variants, largely free from the complicating effects of hybrid immunity from prior natural infection prevalent elsewhere.

In early December 2022, China’s policy shifted abruptly to the full relaxation of control measures, leading to widespread transmission of BA.5, BF.7, and later XBB subvariants [5,6]. Estimating the clinical severity of multiple emerging subvariants, ranging from asymptomatic infection to severe/critical disease and death, is then key for pandemic preparedness and policy evaluation. In China, clinical severity has been classified according to the National Diagnosis and Treatment Protocol (versions 7–10), which aligns with WHO definitions for mild, moderate, severe, and critical cases. However, reported estimates were influenced by multiple factors [7,8,9], including study settings, testing strategies, control measure stringency, demographic characteristics, and the emergence of different Omicron subvariants. For instance, when the government lifted aggressive testing and contact tracing during the post-relaxation period, fewer mild and asymptomatic cases were detected, potentially affecting the apparent severity of infection [10,11,12,13].

To address these complexities, we systematically reviewed and analyzed estimates from studies in China that reported symptoms including fever, cough, or sore throat and the clinical severity of Omicron infections including symptomatic, severe/critical infections, and death to examine the role of epidemiological, demographic, and population-level factors.

## 2. Methods

### 2.1. Search Strategy and Selection Criteria

This systematic review followed the 2020 Preferred Reporting Items for Systematic Review and Meta-analysis (PRISMA) statement in the absence of registration [14]. A systematic search of the literature was conducted on 18 November 2024 in PubMed and China National Knowledge Infrastructure (CNKI), using the terms “((SARS-CoV-2 OR COVID-19) AND (symptom OR severity OR death OR fatality) AND China AND Omicron)” without language restrictions (Supplementary Note S1). To estimate Omicron-associated severity and identify factors driving its outcomes, we included studies published since 1 January 2022. This timeframe aligns with prior studies and surveillance indicating that widespread Omicron transmission became established in early 2022 [6]. Additional relevant articles from the bibliographies of identified articles were also reviewed. Two authors (CW and XH) independently screened the titles and full texts and extracted data from the included studies, coming to a consensus together with the corresponding author (TKT). Studies identified from different databases were de-duplicated.

We included studies that reported ≥1 of the common systemic COVID-19 symptoms or clinical severity of Omicron infections as defined in [15] (Supplementary Note S2) including (1) fever, (2) cough, (3) sore throat, (4) symptomatic infection (all identified cases minus asymptomatic cases), (5) severe/critical illness, and (6) death. We excluded articles where (1) the number of cases was <30, (2) they used imported cases only, (3) the specialized cohort that was not generalizable, such as pregnant women, patients with lung injury or cancer, (4) the full texts were unavailable, or (5) they used non-mainland China settings.

Data extraction used a standardized form (Table S1) including: (1) first author, (2) study period, (3) study location (city and province), (4) case ascertainment methods, (5) study setting, including: (a) Outbreak investigations, defined as studies aimed to identify and follow all reported cases during an outbreak or contact tracing studies that cover all identified primary and secondary cases; (b) Fangcang hospitals, referred to as large-scale, temporary medical facilities rapidly created by converting existing public venues (stadiums or exhibition centers) into healthcare spaces [16]. Implemented first in China during the COVID-19 pandemic, their primary purpose was to isolate patients with mild to moderate infections from communities while providing basic medical care, monitoring, and essential living support; (c) Emergency hospitals, which are designated COVID-19 hospitals (existing hospitals or fever clinics repurposed for pandemic response). Unlike Fangcang hospitals that isolated mild-to-moderate cases, emergency hospitals focused on patients requiring moderate-to-critical medical care. These facilities primarily admitted patients transferred from Fangcang or other overwhelmed hospitals; and (d) Cross-sectional surveys, (6) predominant subvariant, (7) primary or booster vaccination coverage, (8) mean or median age of cases (approximated by a weighted mean of distributions of all age groups if such information was not available), (9) definition of symptom or severity of infection, (10) case counts per each symptom or severity group, (11) total identified cases, (12) COVID-19 antiviral treatment coverage, and (13) proportion of cases with previous infection. Subvariant predominance was determined using study data or genomic surveillance [6,17,18,19]. For studies that did not perform subvariant-specific testing but provided clear and accurate study periods, we inferred the predominant subvariant based on national genomic surveillance and epidemiological reports corresponding to those timeframes. If neither the study-specific subvariant information nor a clear study period was available, the subvariant type was considered missing. For overlapping studies in the same location and during the same period, the largest sample was retained.

### 2.2. Risk of Bias Assessment

Each study was evaluated using the Risk of Bias in Non-randomized Studies-of Interventions (ROBINS-I) [20]. The quality of each study was rated based on the following seven items including: (I) enrolment of all patients satisfying the criteria for inclusion, (II) information on any confounders or efforts to address confounding, (III) clarity of symptom or clinical severity definition, (IV) RT-PCR confirmation, (V) outcome monitored by clinicians/practitioners rather than self-reporting, (VI) loss to follow-up or missing in symptom or severity group of less than 5%, and (VII) accurate period of time when the infection outcome was assessed. A study with any item that is categorized as high risk will be determined to have a high risk of bias (Figures S1 and S2).

### 2.3. Data Analysis

We computed percentages (with 95% confidence intervals (CI)) for each symptom/severity group. The median and interquartile range (IQR) were computed to represent the distribution of reported estimates. Then, we estimated logit-transformed pooled percentages (log-odds) by random-effects meta-analyses using the inverse variance method and maximum likelihood estimator for heterogeneity [21,22]. Results were then back-transformed to percentages for interpretation. Between-study heterogeneity was assessed using Cochran’s *Q* and the *I*^2^ statistic with an *I*^2^ value more than 75% indicating high heterogeneity [23].

Estimates were disaggregated by study settings, age group (categorized by mean or median age), and primary and booster vaccination coverage. We categorized the mean/median age of cases into three group: (1) <18, (2) 18–59, and (3) ≥60 years. Primary or booster vaccination coverage of cases was divided into those <50% or ≥50%. We calculated between-study variance $\tau^{2}$ and computed percent change in $\tau^{2}$ when incorporating each factor as the explained variance in heterogeneity.

Using studies that reported vaccination status-stratified severity estimates, we computed pooled RR for the percentage of cases with symptoms (including the percentage of cases with fever, cough, sore throat, or symptomatic infection) and the percentage of severe/critical infections for vaccinated cases (recipients with at least primary doses) versus the nonvaccinated to estimate the effectiveness of primary and booster doses on preventing systemic symptoms or severe/critical illness in different settings.

### 2.4. Meta-Regression

To evaluate the impact of public health and social measures (PHSMs) on the symptom and clinical severity of COVID-19 infections, we analyzed three key indicators from OxCGRT including CHI, GRI, and SI [24]. These indicators aggregate policy measures across containment, economic, health, vaccination, and miscellaneous domains, scored from 0 to 100 (most stringent). We computed average values in each indicator during the study period. Using meta-regression adjusted for the study setting, age group, predominant subvariant, and vaccination coverage (baseline model), we assessed associations between city-level indicator scores and symptom and clinical severity estimates, with the *p* value < 0.05 indicating statistical significance. Here, we assumed that all indicators were 0 when the full relaxation of control measures were implemented since 11 December 2022 in mainland China [5]. Further, we examined the effects of the complete relaxation of control measures (pre- vs. post-11 December 2022) as another proxy of control intensity and antiviral treatment on the symptoms and severity of infection by applying the same adjustments.

All statistical analyses were conducted using R version 4.4.2 (R Foundation for Statistical Computing, Vienna, Austria). Meta-analysis and meta-regression were conducted by R package “meta” version 8.0 and “metafor” version 4.6-0 using metaprop, metabin, and metareg functions.

## 3. Results

**Figure 1:**
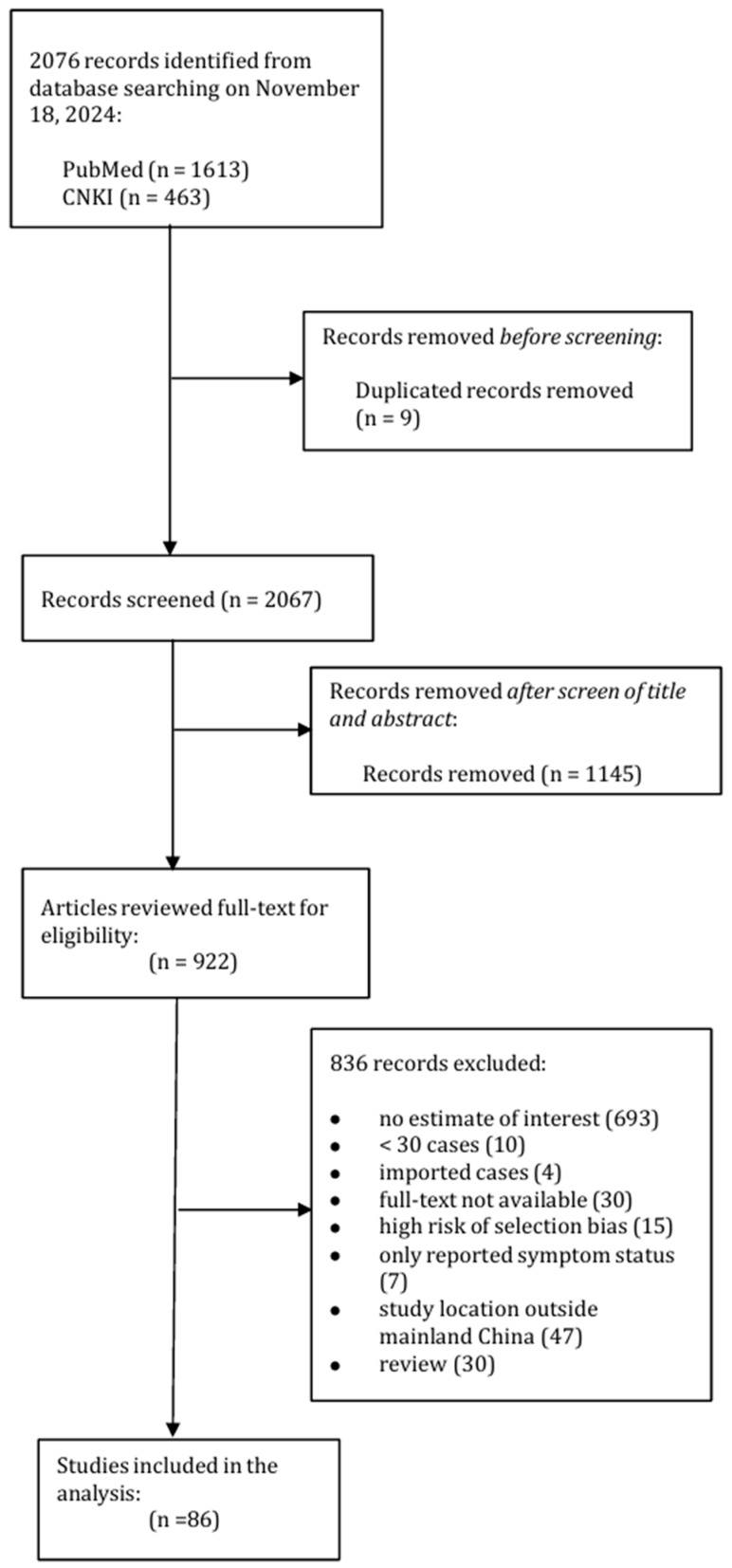
PRISMA flow diagram of searching and screening approach.

In this systematic review, we identified 2076 studies with 9 duplicates removed. A total of 1022 articles remained after screening the titles and abstracts (Figure 1). On the basis of our selection criteria, 86 studies regarding Omicron infection were included in the analysis [8,10,11,12,13,25,26,27,28,29,30,31,32,33,34,35,36,37,38,39,40,41,42,43,44,45,46,47,48,49,50,51,52,53,54,55,56,57,58,59,60,61,62,63,64,65,66,67,68,69,70,71,72,73,74,75,76,77,78,79,80,81,82,83,84,85,86,87,88,89,90,91,92,93,94,95,96,97,98,99,100,101,102,103,104,105]. Overall, 58, 53, and 46 studies provided information on cases with fever, cough, and sore throat (Figures S3–S5), respectively. There were 54, 38, and 20 studies providing the number of symptomatic, severe/critical, and deceased cases (Figures S6–S8). In total, we computed 224 estimates on the symptom and clinical severity of the Omicron infection from included studies. In terms of the predominant subvariant, 11 and 36 studies reported cases for Omicron BA.1 and BA.2 subvariants, 11, 7, and 3 studies reported estimates for BA.5, BF.7, and XBB subvariants, respectively. In addition, six studies were conducted when multiple subvariants were co-circulating. Study characteristics, the number of studies, and computed estimates for each symptom or severity group were summarized in Supplementary Tables S2 and S4.

### 3.1. Symptom and Clinical Severity of Omicron Infections in China

We computed 61, 56, and 47 estimates for the percentage of cases with fever, cough, and sore throat from 58, 53, and 46 studies, which ranged from 7% to 98% (IQR: 28–65%), 6% to 92% (IQR: 36–71%), and 0.5% to 76% (IQR: 16–42%), respectively, for different Omicron subvariant domains. In addition, 61, 40, and 20 estimates for the percentage of symptomatic, severe/critical, and deceased cases from 54, 38, and 20 studies, which ranged from 6% to 100% (IQR: 53–91%), 0% to 63% (IQR: 0–8%), and 0 to 22% (IQR: 0–2%), respectively (Table S4). *I*^2^ values were between 91.7% and 100%, indicating considerable heterogeneity (Figures S3–S8).

### 3.2. Impact of Epidemiological Factors and Effectiveness of Vaccines

**Figure 2:**
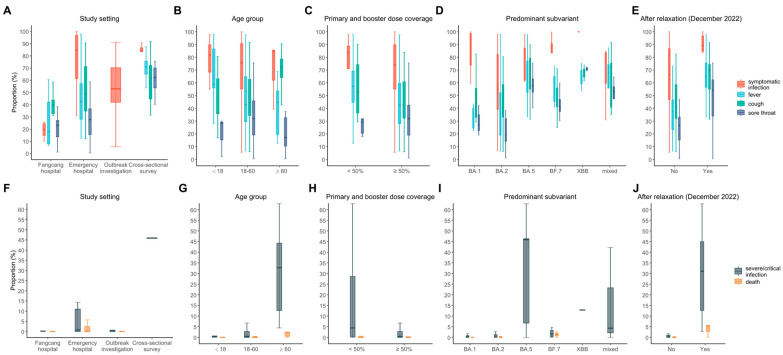
Distributions of the computed percentage of cases with fever, cough, sore throat, symptomatic, severe/critical, and deceased cases, disaggregated by (**A**,**F**) study design, (**B**,**G**) age group, (**C**,**H**) primary and booster vaccination coverage, (**D**,**I**) predominant circulating Omicron subvariants, and (**E**,**J**) whether the study was conducted during the pre- or post-relation period. Boxes indicate interquartile ranges while bold horizontal lines in the boxes represent the median values of computed estimates. Vertical lines give the range (minimums and maximums) of computed estimates by each factor.

**Figure 3:**
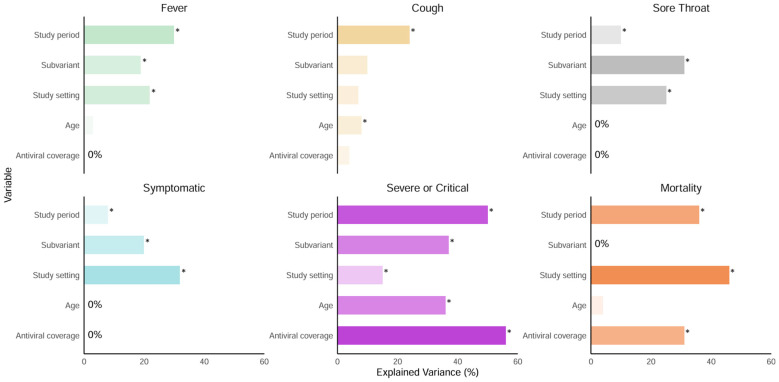
Relative decreases in the heterogeneity in symptom and clinical severity estimates when incorporating epidemiological and demographic variables including age group, antiviral treatment coverage, study period, study setting, and the predominant circulating subvariant into the meta-analysis. The asterisk symbols (*) indicate statistical significance for moderator test (Wald Z-test) with *p*-value < 0.05.

Overall, estimates of Omicron infection severity in China exhibited substantial heterogeneity across study settings, age groups, case vaccination status, predominant subvariant, and temporal periods relative to the relaxation of PHSMs (Figure 2). The analysis of heterogeneity in COVID-19 outcomes reveals distinct contributions of individual factors among clinical and epidemiological features. Study periods emerged as significant drivers of heterogeneity, explaining substantial proportions of variability (8–50%) in all symptom and clinical severity groups (Figure 3, Table S4). The study setting and Omicron subvariant also demonstrated notable impacts on observed heterogeneity in most estimates, though their contributions varied by outcome. Antiviral treatment coverage showed a moderate influence on severe/critical or fatal cases, explaining 56% and 31% heterogeneity, respectively (Figure 3, Table S4). Notably, age explained limited heterogeneity in the estimates of most outcomes (up to 36%), except the percentage of cases with cough and the severe/critical percentage (Figure 3, Table S4).

Multivariate meta-regression that accounted for study setting, subvariant, and age group under the baseline model revealed several key associations. Compared to Fangcang hospitals, studies conducted in emergency hospitals, outbreak investigations, or cross-sectional surveys reported significantly higher odds of symptomatic infection (Table 1). Cases through cross-sectional surveys exhibited 29% (95% CI: 1–65%) higher odds in severe/critical infection than Fangcang hospitals (Table 1). Regarding the predominant subvariant, we found BA.2 cases had significantly lower odds in symptomatic infection than that for BA.1, while BA.5 cases demonstrated a statistically significant increase of approximately 17% in the odds of severe/critical infections compared to BA.1 (Table 1). No significant differences were observed for BA.5, BF.7, XBB, or mixed subvariants concerning symptomatic infection relative to BA.1. Additionally, elderly adults aged > 60 had significantly higher odds in severe or critical infections (odds ratio (OR): 1.28, 95% CI: 1.18–1.40), compared to adults aged 18–59 (Table 1). After accounting for baseline factors, antiviral uptake among cases showed no association with any of the three clinical severity groups (Table 1). However, the non-significant association between antiviral treatments and reduced severity should be interpreted cautiously, as the limited number of studies (17) substantially reduced statistical power to detect clinically meaningful effects.

In terms of symptoms, we observed a higher OR of cases presenting fever from cross-sectional surveys, which was 1.34 (95% CI: 1.04–1.74), than that in Fangcang hospitals (Table 2). Children and adolescents aged < 18 showed higher odds of presenting fever after infection compared to adults: 1.37 (95% CI: 1.13–1.65). Cases of Omicron subvariants BA.5 and BF.7 were associated with 1.30 (95% CI: 1.06–1.59) and 1.24 (95% CI: 1.00–1.53) higher odds in presenting fever, respectively, compared to BA.1 infection (Table 2). Further, cases infected by BA.5 and XBB had 29% (95% CI: 6–59%) and 38% (95% CI: 2–88%) higher odds in presenting sore throat (Table 2). The percentage of cases with cough was comparable across Omicron subvariants. Despite a relatively smaller number of studies, we found higher antiviral coverage was associated with a higher percentage of cases with sore throat after accounting for baseline characters (Table 2).

**Figure 4:**
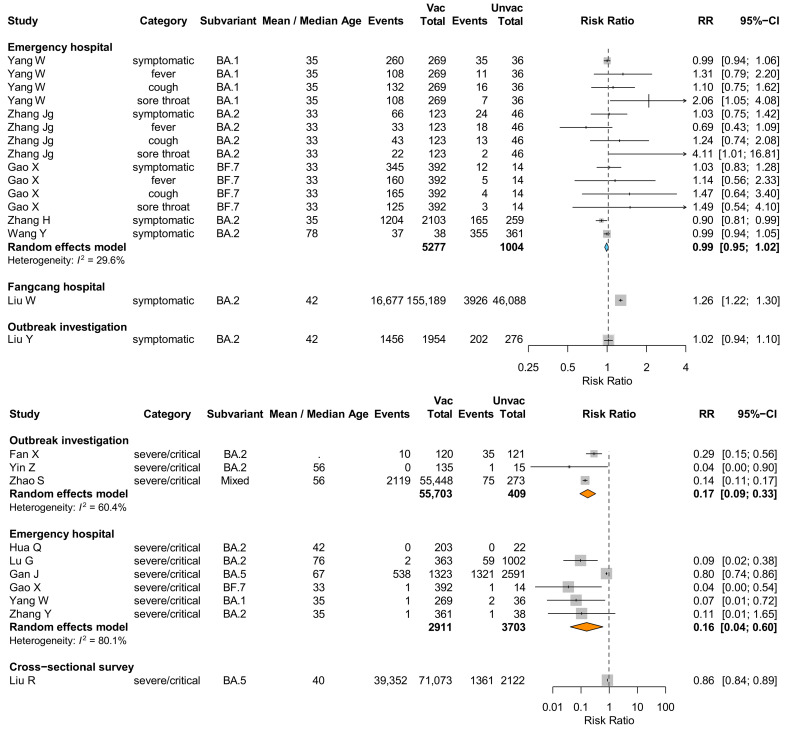
Percentage of cases with symptomatic infection, fever, cough, or sore throat, and the percentage of severe or critical cases for Omicron infections in China for vaccinated cases (at least primary series) compared with nonvaccinated cases. Squares and lines indicate the relative risk (RR) percentage of events of vaccinated cases compared with nonvaccinated cases and the corresponding 95% confidence intervals calculated based on normal approximation. The size of square is proportional to the weight of the study in relation to the pooled estimate. Pooled estimates (blue and orange colored diamonds) were estimated using random-effects meta-analysis. Arrows indicate that 95% confidence intervals are higher than the upper bound of x axis (logarithmic RR).

Based on 19 studies that reported vaccination status-stratified severity estimates for Omicron infections among hospitalized cases, we did not find evidence that primary or booster dose recipients had a lower risk of developing symptoms. The pooled RR for symptomatic infections for cases who received primary doses and a booster dose against nonvaccinated cases in hospitals was 0.99 (95% CI: 0.95–1.02) with a low heterogeneity *<* 30% (Figure 4). In addition, two studies that were conducted in a Fangcang hospital and under an outbreak investigation reported inconsistent estimates where a higher risk of presenting symptoms was associated with primary and booster doses among patients in the Fangcang hospital. In contrast, a significantly lower risk, 83% (95% CI: 67–91%) and 83% (95% CI: 40–96%), of severe/critical infection was found for cases who received primary doses or a booster dose in outbreak investigations and emergency hospitals, compared with nonvaccinated cases, despite an *I*^2^ > 60%, indicating moderate and high heterogeneity.

### 3.3. Role of Government Responses and Control Measures on Estimates of Severity Measures

In multivariate regression analyses adjusted for study setting, age group, and predominant subvariant, we found that each 10-unit increase in GRI or CHI was associated with 7% (95% CI: 1–12%) and 6% (95% CI: 1–10%) lower odds of symptomatic infection, respectively (Table 1). Similarly, each 10-unit increase in GRI, CHI, or SI corresponded to 3% (95% CI: 1–4) lower odds in severe/critical infections (Table 1). Therefore, assuming a baseline symptomatic proportion of 50%, a 10-unit increase in the GRI, CHI, or SI indicator corresponds to an approximate 48% symptomatic proportion. Similarly, a 10-unit increase in GRI, CHI, or SI indicator corresponds to approximately 9.7% of cases who develop severe/critical illness given a 10% baseline percentage of severe/critical cases.

Additionally, the complete relaxation of control measures targeting COVID-19 in mainland China (post-10 December 2022) was associated with 57% (95% CI: 9–126%) and 22% (95% CI: 11–33%) higher odds of symptomatic and severe/critical infections after adjusting for baseline characteristics (Table 1). That is, the percentage of symptomatic and severe/critical cases after the complete relaxation would be 61% (95% CI: 52–69%) and 12% (95% CI: 11–13%) when the baseline percentages of 50% and 10% before relaxation were applied. We also found that the relaxation of PHSMs was associated with 39% (95% CI: 1–90%) higher odds in the percentage of cases with cough (Table 2). However, we did not find relationships between government response indicators and complete relaxation control measures with the percentage of cases with fever, sore throat, or deceased cases (Table 1 and Table 2). These findings were consistent with results from the multivariate regression analysis that further adjusted for the vaccination coverage of cases (Tables S6 and S7).

### 3.4. Risk of Bias

Most studies included in the meta-analysis were judged to be at a moderate risk of bias (fever: 48 (86%) of 57 studies; cough: 44 (85%) of 52 studies; sore throat: 39 (87%) of 45 studies; symptomatic: 37 (88%) of 43 studies; severe/critical: 27 (84%) of 32 studies; and deceased: 17 (89%) of 19 studies. Twelve outbreak investigation studies could not be assessed, Figure S1). Eight (9%) of eighty-six included studies were judged to be at a high risk of bias. The main sources of bias were severity monitored by self-reports rather than clinicians and poor definitions of each symptom or severity group (Figure S2). As a proxy of bias, the language of included articles (English/Chinese) was not associated with the severity estimates, except the percentage of symptomatic cases, where estimates from articles in Chinese were significantly lower than those from articles in English (Table S5). We conducted a sensitivity analysis that removed estimates from studies classified as having serious or critical bias (Table S8) and found similar patterns with estimates in the main analysis.

## 4. Discussion

Accurately characterizing the severity of Omicron infections is critical for informing public health policies. We investigated clinical severity across 86 studies of Omicron in China, focusing on symptom prevalence and severe outcomes. Severity estimates were associated with study settings, subvariants, age, vaccination status, and public health measures, factors that may reflect either real effects on disease or detection biases in different surveillance contexts.

Our findings revealed that studies with higher primary and booster vaccine coverage reported significantly lower risk of severe/critical illness, consistent with multiple studies showing strong protection from inactivated vaccination against severe outcomes (>70%) across Omicron subvariants [106,107,108,109]. In contrast, we did not find evidence that vaccination consistently reduced symptom presence, aligning with previous findings that vaccination offers limited protection against symptomatic Omicron infections, especially for newer subvariants and individuals vaccinated months before infection [110,111]. Our results suggest that in China, protection primarily from inactivated vaccines effectively prevented severe infections but provided minor or no protection against symptoms.

In our meta-regression analysis, higher government response or stringency indices were associated with lower percentages of symptomatic and severe cases. This finding suggests that public health measures had a significant impact on clinical outcomes through reduced viral transmission, preventing healthcare system overwhelm, and ensuring timely care. Behavioral changes during high-response periods may have reduced individuals’ exposure dose, while psychological impacts could have influenced earlier health-seeking behavior [112].

However, associations between public health measures and severity may also reflect detection bias. During stringent control periods, widespread testing identified more asymptomatic and mild cases [113,114,115], naturally lowering the percentage of severe cases. When measures relaxed, testing became less comprehensive, detecting proportionally more symptomatic cases seeking medical attention. Post-relaxation population surveys introduced different biases through self-reporting, where participants with more severe symptoms were likelier to participate, potentially overestimating symptom severity in the general population [116]. It should be noted that our meta-regression analyses have adjusted for the Omicron subtype, reducing the likelihood that differences in viral virulence explain the elevated severity post-relaxation. While biological factors cannot be fully excluded, this adjustment minimizes the case for subtype-driven factors. This suggests non-biological mechanisms, such as shifts in testing practices or healthcare-seeking behavior as mentioned, are more plausible contributors to the observed trend.

Regarding study settings, we observed that the data collection context significantly impacted severity estimates. Emergency hospitals yielded a higher severity compared to Fangcang hospitals and outbreak investigations, as moderate or severe cases were more likely transferred to emergency hospitals, leading to the overestimation of severity. Conversely, Fangcang hospitals primarily admitted mild cases, potentially underestimating the severity of Omicron variant infections [16]. However, several unmeasured confounders such as triage protocols or healthcare disparities related to socio-economic status may have also contributed to the residual heterogeneity in severity [117]. For instance, marginalized groups may delay care or face institutional biases. Additionally, differences in severity classification criteria between emergency and Fangcang hospitals may also drive observed differences.

Although age-related patterns and subvariant differences in Omicron-related severity were not pronounced, we found the percentage of severe/critical cases was significantly higher among adults ≥60 compared to those aged 18–59, consistent with previous findings [118,119,120]. Interestingly, children and adolescents had a higher percentage of fever cases than adults. Individuals infected with later Omicron subvariants (BA.5, BF.7, and XBB) more frequently developed a fever or sore throat compared to those with BA.1, consistent with observations that these variants demonstrate increased fitness in the upper respiratory tract, resulting in more frequent respiratory symptoms [121,122,123]. Consequently, this leads to a higher frequency of respiratory symptoms, including sore throat.

Our study has several limitations. First, our search did not include the Web of Science or Scopus, which may have omitted some studies, particularly from non-biomedical disciplines. However, we believe this effect is minimal given our focused scope on China and the dominance of CNKI in Chinese academic publishing. Expanding database searches could enhance comprehensiveness in broader reviews, which will be prioritized in relevant future work. Second, the small number of studies for certain severity groups (percentage of deceased cases, outbreak investigations) and the limited information on antiviral treatment coverage or previous SARS-CoV-2 exposures may have led to underpowered or false positive estimates (positive relationship with antiviral coverage and percentage of cases with sore throat), though previous exposures were low in pre-relaxation China. Further, heterogeneity in antiviral regimens, administration timing, and dosing protocols across studies likely obscured treatment effects. Compounding this, variability in patient characteristics (e.g., vaccination status, comorbidities) may have also contributed to the observed estimates. These gaps underscore the need for the standardized reporting of antiviral protocols and serological histories in future studies. Third, lacking information on the time since vaccination may underestimate vaccine effectiveness, as protection wanes rapidly after three months since vaccination [124,125]. Despite the potential for variation in time-since-vaccination among participants, we observed consistent VE estimates against symptoms or severe outcomes, which suggests that while waning immunity may contribute to some degree of VE attenuation, the overall effects remain robust. Fourth, minor changes in clinical severity definitions across diagnostic protocols (versions 7–10) could create inconsistent case ascertainment [15], though these likely had limited effects on our results [15]. Finally, we could not evaluate other potential underlying factors due to limited reporting in most studies (Table S3). Critically, this includes unmeasured confounders such as underlying comorbidities and socio-economic determinants (e.g., healthcare access, household density, or occupation) [126]. These factors are known to independently influence COVID-19 severity and vaccination status [127,128]. If unvaccinated individuals disproportionately carried higher comorbidity burdens or socio-economic risk factors, our estimates of VE could be inflated due to residual confounding. Conversely, if vaccinated groups had higher baseline risk profiles, VE might be underestimated. Additionally, adjusting for socio-economic status/residence might reveal that apparent Omicron-related severity differences between populations are partly attributable to structural inequities (e.g., delayed care in rural areas, higher comorbidities in low-socio-economic groups) rather than viral pathogenicity alone [129].

In conclusion, we identified several epidemiological, demographic, and population-level factors that impacted the severity estimates of Omicron infections in China. More recent estimates from studies conducted during the post-relaxation period had significantly higher estimates compared to prior to the relaxation period. There is a need to establish consistent and detailed protocols to provide accurate estimates of severity of SARS-CoV-2 infections in changing epidemiological situations.

## Figures and Tables

**Table 1:** Association of epidemiological and demographic factors and public health measures with clinical severity for Omicron infection in China. Estimates are odds ratios and corresponding 95% confidence intervals.

	Symptomatic Infection	Severe/Critical Infection	Death
Baseline model: adjusted for age group, study setting, and subvariant			
Age group			
0–17	1.10 (0.84, 1.43)	1.04 (0.92, 1.18)	1.00 (0.98, 1.02)
18–59	Reference		
≥60	1.01 (0.78, 1.31)	1.28 (1.18, 1.40) *	1.01 (0.99, 1.03)
Study setting			
Fangcang hospital	Reference		
Emergency hospital	1.57 (1.28, 1.92) *	0.96 (0.81, 1.14)	1.00 (0.98, 1.02)
Outbreak investigation	1.42 (1.12, 1.79) *	0.97 (0.80, 1.17)	-
Cross-sectional survey	1.71 (1.25, 2.34) *	1.29 (1.01, 1.65) *	-
Subvariant			
BA.1	Reference		
BA.2	0.81 (0.67, 0.98) *	0.96 (0.89, 1.04)	1.00 (0.99, 1.02)
BA.5	0.79 (0.62, 1.01)	1.17 (1.05, 1.31) *	-
BF.7	0.92 (0.71, 1.19)	1.02 (0.92, 1.12)	1.01 (0.99, 1.03)
XBB	1.10 (0.71, 1.70)	1.13 (0.94, 1.36)	-
Mixed (any two or more of the above)	0.84 (0.64, 1.09)	1.06 (0.94, 1.19)	-
Baseline model with adjustment for government response index (GRI)			
GRI per 10-unit increase	0.93 (0.88, 0.99) *	0.97 (0.96, 0.99) *	1.00 (0.99, 1.01)
Baseline model with adjustment for containment and health index (CHI)			
CHI per 10-unit increase	0.94 (0.90, 0.99) *	0.97 (0.96, 0.99) *	1.00 (0.99, 1.01)
Baseline model with adjustment for stringency index (SI)			
SI per 10-unit increase	0.96 (0.91, 1.01)	0.97 (0.96, 0.99) *	1.00 (0.99, 1.01)
Baseline model with adjustment for antiviral uptake			
Antiviral coverage ^#^	0.66 (0.21, 2.12)	1.02 (0.98, 1.06)	0.99 (0.98, 1.00)
Baseline model with adjustment for study period			
Before relaxation (before 11 December 2022)	Reference		
After relaxation	1.57 (1.09, 2.26) *	1.22 (1.11, 1.33) *	-

^#^ The numbers of estimates for antiviral treatment coverage are 17, 14, and 10 for symptomatic infection, severe/critical infection, and death, respectively. * Statistically significant, *p* < 0.05.

**Table 2:** Association of epidemiological and demographic factors and public health measures with symptom for Omicron infection in China. Estimates are odds ratios and corresponding 95% confidence intervals.

	Presenting Fever	Presenting Cough	Presenting Sore Throat
Baseline model: adjusted for age group, study setting and subvariant			
Age group			
0–17	1.37 (1.13, 1.65) *	1.05 (0.85, 1.29)	0.97 (0.80, 1.16)
18–59	Reference		
≥60	0.97 (0.82, 1.14)	1.18 (0.99, 1.40)	1.05 (0.90, 1.22)
Study setting			
Fangcang hospital	Reference		
Emergency hospital	1.09 (0.92, 1.30)	1.05 (0.87, 1.28)	1.03 (0.88, 1.21)
Cross-sectional survey	1.34 (1.04, 1.74) *	1.00 (0.76, 1.32)	1.18 (0.93, 1.50)
Subvariant			
BA.1	Reference		
BA.2	1.03 (0.87, 1.21)	0.97 (0.81, 1.16)	0.98 (0.86, 1.13)
BA.5	1.30 (1.06, 1.59) *	1.20 (0.96, 1.51)	1.29 (1.06, 1.59) *
BF.7	1.24 (1.00, 1.53) *	1.05 (0.84, 1.32)	1.17 (0.98, 1.39)
XBB	1.15 (0.80, 1.66)	1.33 (0.90, 1.96)	1.38 (1.02, 1.88) *
Mixed (any two or more of the above)	1.16 (0.89, 1.52)	1.21 (0.91, 1.61)	1.17 (0.93, 1.47)
Baseline model with adjustment for government response index (GRI)			
GRI per 10-unit increase	0.99 (0.95, 1.04)	0.99 (0.94, 1.05)	1.01 (0.97, 1.05)
Baseline model with adjustment for containment and health index (CHI)			
CHI per 10-unit increase	0.99 (0.95, 1.04)	1.00 (0.95, 1.04)	1.01 (0.97, 1.05)
Baseline model with adjustment for stringency index (SI)			
SI per 10-unit increase	1.01 (0.97, 1.06)	1.01 (0.96, 1.06)	1.01 (0.97, 1.05)
Baseline model with adjustment for the antiviral uptake			
Antiviral coverage ^#^	0.86 (0.46, 1.58)	1.99 (0.97, 4.09)	2.03 (1.55, 2.65) *
Baseline model with adjustment for the study period			
Before relaxation (before 11 December 2022)	Reference		
After relaxation	1.16 (0.85, 1.58)	1.39 (1.01, 1.90) *	1.00 (0.79, 1.28)

^#^ The numbers of estimates for antiviral treatment coverage are 20, 16, and 13 for cases presenting fever, cough, and sore throat, respectively. * Statistically significant, *p* < 0.05.

## Data Availability

The data sets generated for this study are available in the supplementary data.

## Supplementary Materials

The following supporting information can be downloaded at: https://www.mdpi.com/article/10.3390/vaccines13070747/s1, Table S1. Extracted variables; Table S2. Summary of included studies; Table S3. Number of computed estimates by severity group; Table S4. Summary of subgroup analysis; Table S5. Sensitivity analysis by language; Table S6. Sensitivity analysis for clinical severity accounting for vaccine coverage; Table S7. Sensitivity analysis for symptoms accounting for vaccine coverage; Table S8. Sensitivity analysis removing critical-risk studies; Figure S1. ROBINS-I scores for each included study rated based on seven items; Figure S2. Overall ratings for all studies based on ROBINS-I assessment; Figures S3–S5. Computed estimates of the percentage of cases with fever, cough and sore throat; Figures S6–S8. Computed estimates of the percentage of symptomatic cases, severe/critical cases and fatal cases; Supplementary data S1–S3. Data extraction form for cases with fever, cough and sore throat; Supplementary data S4–S6. Data extraction form for symptomatic, severe/critical and fatal infections. (Refs. [8,10,11,12,13,15,25,26,27,28,29,30,31,32,33,34,35,36,37,38,39,40,41,42,43,44,45,46,47,48,49,50,51,52,53,54,55,56,57,58,59,60,61,62,63,64,65,66,67,68,69,70,71,72,73,74,75,76,77,78,79,80,81,82,83,84,85,86,87,88,89,90,91,92,93,94,95,96,97,98,99,100,101,102,103,104,105,130]).

## References

[1] Zheng L., Liu S., Lu F. (2023). Impact of National Omicron Outbreak at the end of 2022 on the future outlook of COVID-19 in China. Emerg. Microbes Infect..

[2] Peng L., Huang X., Wang C., Xin H., Cowling B.J., Wu P., Tsang T.K. (2024). Comparative epidemiology of outbreaks caused by SARS-CoV-2 Delta and Omicron variants in China. Epidemiol. Infect..

[3] Burki T. (2022). Dynamic zero COVID policy in the fight against COVID. Lancet Respir. Med..

[4] Tsang T.K., Sullivan S.G., Huang X., Wang C., Wang Y., Nealon J., Yang B., Ainslie K.E.C., Cowling B.J. (2024). Prior infections and effectiveness of SARS-CoV-2 vaccine in test-negative studies: A systematic review and meta-analysis. Am. J. Epidemiol..

[5] Goldberg E.E., Lin Q., Romero-Severson E.O., Ke R. (2023). Swift and extensive Omicron outbreak in China after sudden exit from ‘zero-COVID’ policy. Nat. Commun..

[6] Pan Y., Wang L., Feng Z., Xu H., Li F., Shen Y., Zhang D., Liu W.J., Gao G.F., Wang Q. (2023). Characterisation of SARS-CoV-2 variants in Beijing during 2022: An epidemiological and phylogenetic analysis. Lancet.

[7] Yu C., Fengzhao Z., Hongmei W., Zeyuan L., Yu L., Yuhang G., Rufei S., Qingzhu J., Xiaorong S., Xia W. (2022). The impact of vaccination on patients with COVID-19 during the wave of Omicron in Shanghai. Front. Public Health.

[8] Li Y.C., Ma Z., Zhong H.Y., You H.L. (2022). Clinical characteristics of children with omicron SARS-CoV-2 infection in Changchun, China from march to april 2022: A retrospective study. Front. Pediatr..

[9] Hu C., Liu Y.K., Sun Q.D., Du Z., Fang Y.Q., Guo F., Wang Y.B., He Y., Cen Y., Zeng F. (2022). Clinical characteristics and risk factors for a prolonged length of stay of patients with asymptomatic and mild COVID-19 during the wave of Omicron from Shanghai, China. BMC Infect. Dis..

[10] Liu R., Zhang Y., Ma J., Wang H., Lan Y., Tang X. (2023). Epidemiological features of SARS-CoV-2 Omicron infection under new control strategy: A cross-sectional study of the outbreak since December 2022 in Sichuan, China. BMC Public Health.

[11] Sun Y., Duan Y., Qian J., Qu Y., Wang Y., Fan G., Huang Q., Li Z., Yang W., Feng L. (2024). A Large-Scale Online Survey on Clinical Severity and Associated Risk Factors for SARS-CoV-2 Omicron Infection—China, April-May 2023. China CDC Wkly..

[12] Yu W., Guo Y., Hu T., Liu Y., Fan Q., Guo L., Zheng B., Kong Y., Zhu H., Yu J. (2023). Incidence and severity of SARS-CoV-2 reinfection, a multicenter cohort study in Shanghai, China. J. Med. Virol..

[13] Huo D., Yu T., Shen Y., Pan Y., Li F., Cui S., Lyu B., Liang Z., Zhang D., Yang P. (2023). A Comparison of Clinical Characteristics of Infections with SARS-CoV-2 Omicron Subvariants BF.7.14 and BA.5.2.48—China, October–December 2022. China CDC Wkly..

[14] Page M.J., McKenzie J.E., Bossuyt P.M., Boutron I., Hoffmann T.C., Mulrow C.D., Shamseer L., Tetzlaff J.M., Akl E.A., Brennan S.E. (2021). The PRISMA 2020 statement: An updated guideline for reporting systematic reviews. BMJ.

[15] Wu Y., Feng X., Gong M., Han J., Jiao Y., Li S., Li T., Shen C., Wang H.Y., Yu X. (2023). Evolution and major changes of the diagnosis and treatment protocol for COVID-19 patients in China 2020–2023. Health Care Sci..

[16] Chen S., Zhang Z., Yang J., Wang J., Zhai X., Bärnighausen T., Wang C. (2020). Fangcang shelter hospitals: A novel concept for responding to public health emergencies. Lancet.

[17] Feng Y., Zhao X., Yin Z., Wu C., Chen Z., Nie K., A R., Li L., Niu P., Wang J. (2022). Surveillance and Analysis of SARS-CoV-2 Variant Importation—China, January-June 2022. China CDC Wkly..

[18] Li Y., Du C., Lv Z., Wang F., Zhou L., Peng Y., Li W., Fu Y., Song J., Jia C. (2024). Rapid and extensive SARS-CoV-2 Omicron variant infection wave revealed by wastewater surveillance in Shenzhen following the lifting of a strict COVID-19 strategy. Sci. Total Environ..

[19] Liu P., Xu J. (2023). Genomic surveillance of SARS-CoV-2 in mainland China after ending the zero-COVID policy, December 2022–January 2023. J. Infect..

[20] Sterne J.A., Hernán M.A., Reeves B.C., Savović J., Berkman N.D., Viswanathan M., Henry D., Altman D.G., Ansari M.T., Boutron I. (2016). ROBINS-I: A tool for assessing risk of bias in non-randomised studies of interventions. BMJ.

[21] Borenstein M., Hedges L.V., Higgins J.P., Rothstein H.R. (2010). A basic introduction to fixed-effect and random-effects models for meta-analysis. Res. Synth. Methods.

[22] Veroniki A.A., Jackson D., Bender R., Kuss O., Langan D., Higgins J.P.T., Knapp G., Salanti G. (2019). Methods to calculate uncertainty in the estimated overall effect size from a random-effects meta-analysis. Res. Synth. Methods.

[23] Higgins J.P., Thompson S.G., Deeks J.J., Altman D.G. (2003). Measuring inconsistency in meta-analyses. BMJ.

[24] Hale T., Angrist N., Goldszmidt R., Kira B., Petherick A., Phillips T., Webster S., Cameron-Blake E., Hallas L., Majumdar S. (2021). A global panel database of pandemic policies (Oxford COVID-19 Government Response Tracker). Nat. Hum. Behav..

[25] Ben S., Gao F., Xu Z., Zhang R., Zhang X., Wang N., Zhang M., Hou L. (2024). The role of hematological parameters in asymptomatic and non-severe cases of Omicron variant infection. Virol. J..

[26] Bi X., Zhang Y., Pan J., Chen C., Zheng Y., Wang J., Chen M., Zhou K., Tung T.H., Shen B. (2022). Differences Between Omicron Infections and Fever Outpatients: Comparison of Clinical Manifestations and Initial Routine Hematology Indicators. Infect. Drug Resist..

[27] Bian X.L., Guo Z., Zhang K., Li M.C., Wu Z.M., Jiang Q., Guo M.M., Fan S.N., Chen J.J., Hui L. (2022). Clinical features of children and their family members with family clusters of SARS-CoV-2 Omicron variant infection in Shanghai, China: An analysis of 380 cases. Zhongguo Dang Dai Er Ke Za Zhi = Chin. J. Contemp. Pediatr..

[28] Cao Z., Sun F., Ding H., Tian Z., Cui Y., Yang W., Hu S., Shi L. (2024). A retrospective analysis of the influencing factors of nucleic acid CT value fluctuation in COVID-19 patients infected with Omicron variant virus in Changchun city. Front. Public Health..

[29] Chen B., Shi J., Chen J., Qiu Y. (2023). Clinical characteristics of current COVID-19 rehabilitation outpatients in China. Open Med..

[30] Chen X., Wang H., Ai J., Shen L., Lin K., Yuan G., Sheng X., Jin X., Deng Z., Xu J. (2022). Identification of CKD, bedridden history and cancer as higher-risk comorbidities and their impact on prognosis of hospitalized Omicron patients: A multi-centre cohort study. Emerg. Microbes Infect..

[31] Cheng L.l., Li Z.t., Wu H.k., Li F., Qiu Y., Wang T., Peng H., Liu Z.h., Huang P.r., Zhou L. (2024). Clinical and pathogen features of COVID-19-associated infections during an Omicron strain outbreak in Guangzhou, China. Microbiol. Spectr..

[32] Chu J., Dai Q., Dong C., Kong X., Tian H., Li C., Peng J., Xu K., Ju H., Bao C. (2024). The serological IgG and neutralizing antibody of SARS-CoV-2 omicron variant reinfection in Jiangsu Province, China. Front. Public Health.

[33] Chu J., Hua L., Liu X., Xiong H., Jiang F., Zhou W., Wang L., Xue G. (2024). Superoxide dismutase alterations in COVID-19: Implications for disease severity and mortality prediction in the context of omicron variant infection. Front. Immunol..

[34] Deng H., Mai Y., Liu H., Guan J. (2023). Clinical characteristics of liver injury in SARS-CoV-2 Omicron variant- and Omicron subvariant-infected patients. Ann. Hepatol..

[35] Du Y., Li C., Zhao W., Li J., Zhao L., Guo H., Jiang Y., Liu W.V., Zeng S., Zhang H. (2024). Multimodal neuroimaging exploration of the mechanisms of sleep quality deterioration after SARS-CoV-2 Omicron infection. BMC Med..

[36] Fan X., Lu S., Bai L., Liu H., Fu J., Jin X., He Y., Lu J., Dong X. (2022). Preliminary Study of the Protectiveness of Vaccination Against the COVID-19 in the Outbreak of VOC Omicron BA.2—Jilin City, Jilin Province, China, March 3–April 12, 2022. China CDC Wkly..

[37] Feng L., Liu X., Zhang L. (2022). Clinical observation of Xuanfei Baidu Granule in the treatment of COVID-19 (Omicron). Tianjin J. Tradit. Chin. Med..

[38] Feng L., Wang X., Li L., Wu Q. (2022). Comparison in clinical characteristics of native and imported people infected with the SARS-CoV-2 Omicron variant. Shandong Med. J..

[39] Feng Y., Shao H., Gong X., Song Z., Xie Y., Qi S., Shi L., Hu Y., Liu X., Liu X. (2022). ‘Dynamic zero-COVID’ policy and viral clearance during an omicron wave in Tianjin, China: A city-wide retrospective observational study. BMJ Open.

[40] Fu J., Cui K., Li Z., Xie Z., Tian H., Liao Y., Gong X., Liu H. (2023). Epidemiological characteristics analysis of the SARS-CoV-2 Omicron variant infection in Gannan area. Infect. Dis. Inf..

[41] Fu J., Mao X., Jiang L. (2022). Relevant Investigation and Physical Analysis of Patients with Persistently Positive of COVID-19 Omicron Variant. J. Emerg. Tradit. Chin. Med..

[42] Fu Z., Liang D., Zhang W., Shi D., Ma Y., Wei D., Xi J., Yang S., Xu X., Tian D. (2023). Host protection against Omicron BA.2.2 sublineages by prior vaccination in spring 2022 COVID-19 outbreak in Shanghai. Front. Med..

[43] Gan J., Zhang H., Wu J., Liu Y., Liu P., Cheng R., Tang X., Yang L., Luo W., Li W. (2024). Effect of inactivated vaccine boosters against severe and critical COVID-19 during the Omicron BA.5 wave: A retrospective analysis of hospitalized patients in China. J. Med. Virol..

[44] Gao M., Xing X., Hao W., Zhang X., Zhong K., Lu C., Deng X., Yu L. (2024). Diverse immune responses in vaccinated individuals with and without symptoms after omicron exposure during the recent outbreak in Guangzhou, China. Heliyon.

[45] Gao X., Wang F., Liu H., Chai J., Tian G., Yao L., Chen C., Huo P., Yao Y., Wen J. (2024). BF. 7: A new Omicron subvariant characterized by rapid transmission. Clin. Microbiol. Infect..

[46] Gong X., Peng L., Wang F., Liu J., Tang Y., Peng Y., Niu S., Yin J., Guo L., Lu H. (2024). Repeated Omicron infection dampens immune imprinting from previous vaccination and induces broad neutralizing antibodies against Omicron sub-variants. J. Infect..

[47] Gu B., Yao L., Zhu X.Y., Zou T., Feng Y.J., Yan J.Y., Zhang J.P., Tang P.J., Chen C. (2022). Comparison of initial clinic characteristics of hospitalized patients in Suzhou City during the COVID-19 Omicron wave with ancestral variant wave. Ther. Adv. Respir. Dis..

[48] Gu Q., Cao Z., Zhang Y. (2022). Clinical analysis of 89 children infected with SARS-CoV-2 in Lianyungang. J. Xuzhou Med. Univ..

[49] Han X., Chen J., Chen L., Jia X., Fan Y., Zheng Y., Alwalid O., Liu J., Li Y., Li N. (2023). Comparative Analysis of Clinical and CT Findings in Patients with SARS-CoV-2 Original Strain, Delta and Omicron Variants. Biomedicines.

[50] He W., Yu F., Wei Y., Sun W., Ren D., Wu Z., Feng Y., Ji N., Wang X., Huang M. (2022). Epidemiology of infections with SARS-CoV-2 Omicron variant in Jiangsu Province, China. J. Nanjing Med. Univ. (Nat. Sci.).

[51] He X., Liao Y., Liang Y., Yu J., Gao W., Wan J., Liao Y., Su J., Zou X., Tang S. (2023). Transmission characteristics and inactivated vaccine effectiveness against transmission of the SARS-CoV-2 Omicron BA.2 variant in Shenzhen, China. Front. Immunol..

[52] Hu C., Xu P., Xu L., Zhang Y., Zhou J., Wang L., Zhou W., Ye L., Lu C. (2022). Short-term persistent symptoms in preschool children with mild/common coronavirus disease 2019 caused by Omicron variant infection after discharge:a follow-up study. Chin. J. Contemp. Pediatr..

[53] Hua Q., Zheng D., Yu B., Tan X., Chen Q., Wang L., Zhang J., Liu Y., Weng H., Cai Y. (2022). Effectiveness of Inactivated COVID-19 Vaccines against COVID-19 Caused by the SARS-CoV-2 Delta and Omicron Variants: A Retrospective Cohort Study. Vaccines.

[54] Li H., Jia X., Wang Y., Lv Y., Wang J., Zhai Y., Xue X. (2023). Differences in the severity and mortality risk factors for patients hospitalized for COVID-19 pneumonia between the early wave and the very late stage of the pandemic. Front. Med..

[55] Li H., Zhu M., Zhang P., Yan X., Niu J., Wang Z., Cao J. (2022). Milder symptoms and shorter course in patients with re-positive COVID-19: A cohort of 180 patients from Northeast China. Front. Microbiol..

[56] Li H., Zhu X., Yu R., Qian X., Huang Y., Chen X., Lin H., Zheng H., Zhang Y., Lin J. (2022). The effects of vaccination on the disease severity and factors for viral clearance and hospitalization in Omicron-infected patients: A retrospective observational cohort study from recent regional outbreaks in China. Front. Cell Infect. Microbiol..

[57] Li J., Song R., Yuan Z., Xu Z., Suo L., Wang Q., Li Y., Gao Y., Li X., Chen X. (2022). Protective Effect of Inactivated COVID-19 Vaccines against Progression of SARS-CoV-2 Omicron and Delta Variant Infections to Pneumonia in Beijing, China, in 2022. Vaccines.

[58] Li Q., Liu X., Li L., Hu X., Cui G., Sun R., Zhang D., Li J., Li Y., Zhang Y. (2022). Comparison of clinical characteristics between SARS-CoV-2 Omicron variant and Delta variant infections in China. Front. Med..

[59] Li Q., Wang Y., Liu H., Peng H., Xiang J., Guo S. (2023). Imaging Progression Under Low Respiratory Viral Load of SARS-CoV-2 Omicron Variant Infection: A Retrospective Study in China. Infect. Drug Resist..

[60] Li T., Han M., Wang J., Zhou C., Mu H. (2022). Clinical characteristics and risks of the convalescent COVID-19 patients with re-detectable positive RNA test: A 430 patients with Omicron infected cross-sectional survey in Tianjin, China. J. Infect. Public Health.

[61] Li X., Wu L., Qu Y., Cao M., Feng J., Huang H. (2022). Clinical characteristics and vaccine effectiveness against SARS-CoV-2 Omicron subvariant BA.2 in the children. Signal Transduct. Target. Ther..

[62] Liu D., Feng S., Sha F., Liao Y., Xie X., Huang F., Kong D., Zhang Z., Chen Z., Chen N. (2023). Inactivated SARS-CoV-2 Vaccine Booster Against Omicron Infection Among Quarantined Close Contacts. JAMA Netw. Open.

[63] Liu L., Zhang J., Zhu H., Pan B., Ma S., Li M., Ma B., Zhou H., Zhang G. (2022). Distribution of Traditional Chinese Medicine Constitution Types and Prevention and Control Strategies in Patients Infected with the Omicron Variant of Severe Acute Respiratory Syndrome Coronavirus 2 in Shanghai, China:An Analysis of 220 Cases. J. Anhui Univ. Chin. Med..

[64] Liu W., Gong F., Zheng X., Pei L., Wang X., Yang S., Zhao S., Yang Z., Lin J., Jing F. (2023). Factors associated with prolonged viral shedding of SARS-CoV-2 Omicron variant infection in Shanghai: A multicenter, retrospective, observational study. J. Med. Virol..

[65] Liu X., Zhang P., Chen M., Zhou H., Yue T., Xu M., Cai T., Huang J., Yue X., Li G. (2023). Epidemiological and clinical features of COVID-19 inpatients in Changsha, China: A retrospective study from 2020 to 2022. Heliyon.

[66] Liu Y., Chai Y.H., Wu Y.F., Zhang Y.W., Wang L., Yang L., Shi Y.H., Wang L.L., Zhang L.S., Chen Y. (2023). Risk factors associated with indoor transmission during home quarantine of COVID-19 patients. Front. Public Health.

[67] Lu G., Zhang Y., Zhang H., Ai J., He L., Yuan X., Bao S., Chen X., Wang H., Cai J. (2022). Geriatric risk and protective factors for serious COVID-19 outcomes among older adults in Shanghai Omicron wave. Emerg. Microbes Infect..

[68] Lv Y., Liu D., Li J. (2022). Influencing factors of nucleic acid negative conversion in patients with asymptomatic and mild COVID-19 induced by the Omicron variant of SARS-CoV-2. Shaanxi Med. J..

[69] Miao Y., Ren Y., Ren T. (2023). Clinical Characteristics Profile of COVID-19 Patients with Omicron Variant Admitted in a Tertiary Hospital, China. Int. J. Gen. Med..

[70] Peng H., Xiang T., Xu F., Jiang Y., Zhong L., Peng Y., Le A., Zhang W., Liu Y. (2023). Redistribution and Activation of CD16brightCD56dim NK Cell Subset to Fight against Omicron Subvariant BA.2 after COVID-19 Vaccination. Microorganisms.

[71] Qin S., Li Y., Wang L., Zhao X., Ma X., Gao G.F. (2023). Assessment of vaccinations and breakthrough infections after adjustment of the dynamic zero-COVID-19 strategy in China: An online survey. Emerg. Microbes Infect..

[72] Qin T., Zheng X., Feng J., Lu W., Zhou K., Ling Y., Qin Q., Zou D., Zhang J., Lu J. (2023). Risk Factors and Their Influence Analysis on Mortality of 858 COVID-19 Pneumonia Patients with Omicron Variant. GuangXi Sci..

[73] Qiu W., Shi Q., Chen F., Wu Q., Yu X., Xiong L. (2022). The derived neutrophil to lymphocyte ratio can be the predictor of prognosis for COVID-19 Omicron BA.2 infected patients. Front. Immunol..

[74] Qu L., Xie C., Qiu M., Yi L., Liu Z., Zou L., Hu P., Jiang H., Lian H., Yang M. (2024). Characterizing Infections in Two Epidemic Waves of SARS-CoV-2 Omicron Variants: A Cohort Study in Guangzhou, China. Viruses.

[75] Sha J., Meng C., Sun J., Sun L., Gu R., Liu J., Zhu X., Zhu D. (2023). Clinical and upper airway characteristics of 3715 patients with the Omicron variant of SARS-CoV-2 in Changchun, China. J. Infect. Public Health.

[76] Shao J., Fan R., Hu J., Zhang T., Lee C., Huang X., Wang F., Liang H., Jin Y., Jiang Y. (2022). Clinical Progression and Outcome of Hospitalized Patients Infected with SARS-CoV-2 Omicron Variant in Shanghai, China. Vaccines.

[77] Shen J., Wu L., Wang P., Shen X., Jiang Y., Liu J., Chen W. (2022). Clinical characteristics and short-term recovery of hyposmia in hospitalized non-severe COVID-19 patients with Omicron variant in Shanghai, China. Front. Med..

[78] Shen N., Wu Y.F., Chen Y.W., Fang X.Y., Zhou M., Wang W.Y., Tang M.Y., Pan Q.H., Ma J., Zhang H. (2023). Clinical characteristics of pediatric cases infected with the SARS-CoV-2 Omicron variant in a tertiary children’s medical center in Shanghai, China. World J. Pediatr..

[79] Shen X., Wang P., Shen J., Jiang Y., Wu L., Nie X., Liu J., Chen W. (2023). Neurological Manifestations of hospitalized patients with mild to moderate infection with SARS-CoV-2 Omicron variant in Shanghai, China. J. Infect. Public Health.

[80] Shen Y., Ai J., Lin N., Zhang H., Li Y., Wang H., Wang S., Wang Z., Li T., Sun F. (2022). An open, prospective cohort study of VV116 in Chinese participants infected with SARS-CoV-2 omicron variants. Emerg. Microbes Infect..

[81] Su Z., Li Y., Xie Y., Huang Z., Cheng A., Zhou X., Li J., Qin R., Wei X., Liu Y. (2024). Acute and long COVID-19 symptoms and associated factors in the omicron-dominant period: A nationwide survey via the online platform Wenjuanxing in China. BMC Public Health.

[82] Sun F., Zhang Y., Li Y., Zhang H., Liu Q., Ai J., Wang S., Cui S., Shi L., Xue Y. (2022). Inactivated vaccine protects against severe outcome among adults aged over 60 years during SARS-CoV-2 Omicron variant predominance:evidence from a single-center cohort. J. Microbes Infect..

[83] Sun H., Zhang Y., Shi C., Liu X., Zhao G., Zhao Q., Chen M., Wu Z., Lv S., Zhao X. (2022). Factor analysis of nucleic acid turning negative time of novel coronavirus Omicron infection patients treated by integrated traditional Chinese and Western medicine in Tianjin area. Tianjin J. Tradit. Chin. Med..

[84] Tong X., Yang Y., Hu W. (2023). Immune response patterns of patients with novel coronavirus pneumonia. Zhejiang Clin. Med..

[85] Wang K., Guo Z., Zeng T., Sun S., Lu Y., Wang J., Li S., Luan Z., Li H., Zhang J. (2023). Transmission Characteristics and Inactivated Vaccine Effectiveness Against Transmission of SARS-CoV-2 Omicron BA.5 Variants in Urumqi, China. JAMA Netw. Open.

[86] Wang Y., Yu G., Shi J., Zhang X., Huo J., Li M., Chen J., Yu L., Li Y., Han Z. (2024). Retrospective study about clinical severity and epidemiological analysis of the COVID-19 Omicron subvariant lineage-infected patients in Hohhot, China. BMC Infect. Dis..

[87] Wang Y., Zhao D., Chen X., Liu X., Xiao W., Feng L. (2023). The effect of nirmatrelvir-ritonavir on viral clearance and length of hospital stay in patients infected with SARS-CoV-2 omicron variants. Influenza Other Respir Viruses.

[88] Wang Z., Liu B., Qi X., Zhang R., Bian S., Jiang M. (2022). Epidemiological characteristics of local COVID-19 cases in Zhejiang Province. Prev. Med..

[89] Wei X., Fang Z., Zhu H., Gan C., Huang M. (2022). Myocardium damage and electrocardiogram characteristics of patients infected with SARS-CoV-2 Omicron variant in Zhuhai. Chin. J. Arterioscler..

[90] Wu D., Ye Y., Tang L., Wang A.B., Zhang R., Qian Z.H., Wang F.Z., Zheng H., Huang C., Lv X.Y. (2022). A case-case study on the effect of primary and booster immunization with China-produced COVID-19 vaccines on prevention of pneumonia and viral load among vaccinated persons infected by Delta and Omicron variants. Emerg. Microbes Infect..

[91] Xian L., Lin J., Yu S., Zhao Y., Zhao P., Cao G. (2022). Epidemiological characteristics of SARS-CoV-2 infection outbreak in Shanghai in the Spring of 2022. Shanghai J. Prev. Med..

[92] Xu T., Chen Y., Zhan W., Chung K.F., Qiu Z., Huang K., Chen R., Xie J., Wang G., Zhang M. (2024). Profiles of Cough and Associated Risk Factors in Nonhospitalized Individuals With SARS-CoV-2 Omicron Variant Infection: Cross-Sectional Online Survey in China. JMIR Public Health Surveill.

[93] Xu X., Zhou S., Chen C., Li J., Wu H., Jin G., Zhou J., Wang G., Cao M., Sun D. (2023). Efficacy and safety of Reyanning mixture in patients infected with SARS-CoV-2 Omicron variant: A prospective, open-label, randomized controlled trial. Phytomedicine.

[94] Zhang Y., Xie X., Yu X., Liang H., Liu K., Li L., Wan Y., Wang J. (2022). Epidemiological characteristics of SARS-CoV-2 Omicron variant patients in a Shanghai Fangcang hospital. Infect. Dis. Inf..

[95] Yang H., Wang Z., Zhang Y., Xu M., Wang Y., Zhang Y., Liu X., An Z., Tong Z. (2023). Clinical characteristics and factors for serious outcomes among outpatients infected with the Omicron subvariant BF.7. J. Med. Virol..

[96] Yang N., Wang C., Huang J., Dong J., Ye J., Fu Y., Huang J., Xu D., Cao G., Qian G. (2022). Clinical and Pulmonary CT Characteristics of Patients Infected With the SARS-CoV-2 Omicron Variant Compared With Those of Patients Infected With the Alpha Viral Strain. Front. Public Health.

[97] Yang W., Yang S., Wang L., Zhou Y., Xin Y., Li H., Mu W., Wu Q., Xu L., Zhao M. (2022). Clinical characteristics of 310 SARS-CoV-2 Omicron variant patients and comparison with Delta and Beta variant patients in China. Virol Sin..

[98] Yang Y., Guo L., Yuan J., Xu Z., Gu Y., Zhang J., Guan Y., Liang J., Lu H., Liu Y. (2023). Viral and antibody dynamics of acute infection with SARS-CoV-2 omicron variant (B.1.1.529): A prospective cohort study from Shenzhen, China. Lancet Microbe..

[99] Yin Z., Fang Q., Wen T., Zheng C., Fu C., Wang S., Li J., Gong X. (2023). Effectiveness of COVID-19 vaccines against SARS-CoV-2 Omicron variants during two outbreaks from March to May 2022 in Quzhou, China. Hum. Vaccine Immunother..

[100] Ying-Hao P., Yuan-Yuan G., Hai-Dong Z., Qiu-Hua C., Xue-Ran G., Hai-Qi Z., Hua J. (2022). Clinical characteristics and analysis of risk factors for disease progression of patients with SARS-CoV-2 Omicron variant infection: A retrospective study of 25207 cases in a Fangcang hospital. Front. Cell Infect. Microbiol..

[101] Zeng Q.L., Lv Y.J., Liu X.J., Jiang Z.Y., Huang S., Li W.Z., Yu Z.J. (2022). Clinical Characteristics of Omicron SARS-CoV-2 Variant Infection After Non-mRNA-Based Vaccination in China. Front. Microbiol..

[102] Zhang H., Weng Z., Zheng Y., Zheng M., Chen W., He H., Ye X., Zheng Y., Xie J., Zheng K. (2023). Epidemiological and clinical features of SARS-CoV-2 Omicron variant infection in Quanzhou, Fujian province: A retrospective study. Sci. Rep..

[103] Zhang J., Chen N., Zhao D., Zhang J., Hu Z., Tao Z. (2022). Clinical Characteristics of COVID-19 Patients Infected by the Omicron Variant of SARS-CoV-2. Front. Med..

[104] Zhang K., Zhong X., Fan X., Yu D., Chen Z., Zhao C., Zhang X., Guan Z., Wei X., Wan S. (2024). Asymptomatic infection and disappearance of clinical symptoms of COVID-19 infectors in China 2022-2023: A cross-sectional study. Sci. Rep..

[105] Zhao S., Luo K., Guo Y., Fang M., Sun Q., Dai Z., Yang H., Zhan Z., Hu S., Chen T. (2023). Analysis of Factors Influencing the Clinical Severity of Omicron and Delta Variants. Trop. Med. Infect. Dis..

[106] Tang L., Wang F.Z., Rodewald L.E., Wang X.Y., Liu S.Y., Liu Q.Q., Wang X.Q., Wu D., Li M.S., Zhang Q. (2023). Real-World Effectiveness of Primary Series and Booster Doses of Inactivated Coronavirus Disease 2019 Vaccine Against Omicron BA.2 Variant Infection in China: A Retrospective Cohort Study. J. Infect. Dis..

[107] McMenamin M.E., Nealon J., Lin Y., Wong J.Y., Cheung J.K., Lau E.H.Y., Wu P., Leung G.M., Cowling B.J. (2022). Vaccine effectiveness of one, two, and three doses of BNT162b2 and CoronaVac against COVID-19 in Hong Kong: A population-based observational study. Lancet Infect. Dis..

[108] Wei Y., Jia K.M., Zhao S., Hung C.T., Mok C.K.P., Poon P.K.M., Man Leung E.Y., Wang M.H., Yam C.H.K., Chow T.Y. (2023). Estimation of Vaccine Effectiveness of CoronaVac and BNT162b2 Against Severe Outcomes Over Time Among Patients With SARS-CoV-2 Omicron. JAMA Netw. Open.

[109] Zhao D., Sun Y., Li J., Li X., Ma Y., Cao Z., Zhang J., Ma J., Li J., Wang Q. (2024). Effectiveness of inactivated COVID-19 vaccines in preventing COVID-19-related hospitalization during the Omicron BF.7-predominant epidemic wave in Beijing, China: A cohort study. BMC Infect. Dis..

[110] Xu H., Li H., You H., Zhang P., Li N., Jiang N., Cao Y., Qin L., Qin G., Qu H. (2023). Effectiveness of inactivated COVID-19 vaccines against mild disease, pneumonia, and severe disease among persons infected with SARS-CoV-2 Omicron variant: Real-world study in Jilin Province, China. Emerg. Microbes Infect..

[111] Tsang N.N.Y., So H.C., Cowling B.J., Leung G.M., Ip D.K.M. (2023). Effectiveness of BNT162b2 and CoronaVac COVID-19 vaccination against asymptomatic and symptomatic infection of SARS-CoV-2 omicron BA.2 in Hong Kong: A prospective cohort study. Lancet Infect. Dis..

[112] Purcell H., Kohler I.V., Ciancio A., Mwera J., Delavande A., Mwapasa V., Kohler H.P. (2024). Mortality risk information and health-seeking behavior during an epidemic. Proc. Natl. Acad. Sci. USA.

[113] Shi Q., Hu Y., Peng B., Tang X.J., Wang W., Su K., Luo C., Wu B., Zhang F., Zhang Y. (2021). Effective control of SARS-CoV-2 transmission in Wanzhou, China. Nat. Med..

[114] Hellewell J., Russell T.W., Beale R., Kelly G., Houlihan C., Nastouli E., Kucharski A.J. (2021). Estimating the effectiveness of routine asymptomatic PCR testing at different frequencies for the detection of SARS-CoV-2 infections. BMC Med..

[115] Shental N., Levy S., Wuvshet V., Skorniakov S., Shalem B., Ottolenghi A., Greenshpan Y., Steinberg R., Edri A., Gillis R. (2020). Efficient high-throughput SARS-CoV-2 testing to detect asymptomatic carriers. Sci. Adv..

[116] Shang W., Kang L., Cao G., Wang Y., Gao P., Liu J., Liu M. (2022). Percentage of Asymptomatic Infections among SARS-CoV-2 Omicron Variant-Positive Individuals: A Systematic Review and Meta-Analysis. Vaccines.

[117] Karmakar M., Lantz P.M., Tipirneni R. (2021). Association of Social and Demographic Factors With COVID-19 Incidence and Death Rates in the US. JAMA Netw. Open.

[118] Wolter N., Jassat W., Walaza S., Welch R., Moultrie H., Groome M., Amoako D.G., Everatt J., Bhiman J.N., Scheepers C. (2022). Early assessment of the clinical severity of the SARS-CoV-2 omicron variant in South Africa: A data linkage study. Lancet.

[119] Wong J.Y., Cheung J.K., Lin Y., Bond H.S., Lau E.H.Y., Ip D.K.M., Cowling B.J., Wu P. (2023). Intrinsic and Effective Severity of Coronavirus Disease 2019 Cases Infected With the Ancestral Strain and Omicron BA.2 Variant in Hong Kong. J. Infect. Dis..

[120] Chen X., Yan X., Sun K., Zheng N., Sun R., Zhou J., Deng X., Zhuang T., Cai J., Zhang J. (2022). Estimation of disease burden and clinical severity of COVID-19 caused by Omicron BA.2 in Shanghai, February-June 2022. Emerg. Microbes Infect..

[121] Nakakubo S., Kishida N., Okuda K., Kamada K., Iwama M., Suzuki M., Yokota I., Ito Y.M., Nasuhara Y., Boucher R.C. (2023). Associations of COVID-19 symptoms with omicron subvariants BA.2 and BA.5, host status, and clinical outcomes in Japan: A registry-based observational study. Lancet Infect. Dis..

[122] Wickenhagen A., Flagg M., Port J.R., Yinda C.K., Goldin K., Gallogly S., Schulz J.E., Lutterman T., Williamson B.N., Kaiser F. (2025). Evolution of Omicron lineage towards increased fitness in the upper respiratory tract in the absence of severe lung pathology. Nat. Commun..

[123] Shuai H., Chan J.F., Hu B., Chai Y., Yoon C., Liu H., Liu Y., Shi J., Zhu T., Hu J.C. (2023). The viral fitness and intrinsic pathogenicity of dominant SARS-CoV-2 Omicron sublineages BA.1, BA.2, and BA.5. EBioMedicine.

[124] Menni C., May A., Polidori L., Louca P., Wolf J., Capdevila J., Hu C., Ourselin S., Steves C.J., Valdes A.M. (2022). COVID-19 vaccine waning and effectiveness and side-effects of boosters: A prospective community study from the ZOE COVID Study. Lancet Infect. Dis..

[125] Stowe J., Andrews N., Kirsebom F., Ramsay M., Bernal J.L. (2022). Effectiveness of COVID-19 vaccines against Omicron and Delta hospitalisation, a test negative case-control study. Nat. Commun..

[126] Russell C.D., Lone N.I., Baillie J.K. (2023). Comorbidities, multimorbidity and COVID-19. Nat. Med..

[127] Magesh S., John D., Li W.T., Li Y., Mattingly-App A., Jain S., Chang E.Y., Ongkeko W.M. (2021). Disparities in COVID-19 Outcomes by Race, Ethnicity, and Socioeconomic Status: A Systematic-Review and Meta-analysis. JAMA Netw. Open.

[128] Mena G.E., Martinez P.P., Mahmud A.S., Marquet P.A., Buckee C.O., Santillana M. (2021). Socioeconomic status determines COVID-19 incidence and related mortality in Santiago, Chile. Science.

[129] Jafari A.S., Mozaffari Nejad A.S., Faraji H., Abdel-Moneim A.S., Asgari S., Karami H., Kamali A., Kheirkhah Vakilabad A.A., Habibi A., Faramarzpour M. (2025). Diagnostic Challenges in Fungal Coinfections Associated With Global COVID-19. Scientifica.

[130] Guddat C., Grouven U., Bender R., Skipka G. (2012). A note on the graphical presentation of prediction intervals in random-effects meta-analyses. Syst Rev..

