# Supplementary material for: Impact of Vaccination and Public Health Measures on the Severity of SARS-CoV-2 Omicron Infections in China: A Systematic Review and Meta-Regression Analysis
Source: Vaccines (Basel). 2025 Jul 12;13(7):747. doi: 10.3390/vaccines13070747 (PMC12301040; doi:10.3390/vaccines13070747)
Supplement: Supplementary file 1 [file vaccines-13-00747-s001.zip › severity_China_appendix.pdf]

Supplementary information

for ‘’ Impact of Vaccination and Public Health Measures on Omicron Severity in China: A Systematic Review and Meta-regression Analysis”

Supplementary Note 1: Full search terms

PubMed

(January 1, 2022 to November 18, 2024), 1613 articles

(SARS-CoV-2 OR COVID-19) AND (symptom OR severity OR death OR fatality) AND China AND Omicron

China National Knowledge Infrastructure (CNKI) (URL: https://www.cnki.net/index/)

(January 1, 2022 to November 18, 2024), 463 articles

FT = (新型冠状病毒 or COVID-19) and FT = (症状 or 临床分型 or 死亡 or 住院) and FT = (Omicron or 奥密克戎)

新型冠状病毒: SARS-CoV-2

症状: Symptom

临床分型: Clinical severity

死亡: Death

住院: Hospitalization

奥密克戎: Omicron

Supplementary Note 2: Definitions of case severity of COVID-19 in China

Most studies included in our analysis followed the Diagnosis and Treatment Protocol for COVID-19 patients (versions 7-10) that was issued through March 2020 to January 2023. The differences among these versions including transmission characteristics across different variants, testing strategies, vaccination recommendations and supplementary ascertainment methods using serology or antigen tests [1]. Clinical classifications remain similar across version 7 to version 10 [1].

According to the Diagnosis and Treatment Protocol for COVID-19 patients, asymptomatic infection was defined as: having positive results from viral nucleic acid or antibody testing with no relevant clinical manifestations including fever, cough, fatigue, sore throat, loss of taste or smell, diarrhea, and no imaging characteristics of COVID-19. In our analysis, symptomatic cases were categorized as the number of all cases minus the number of asymptomatic cases.

Mild cases were those who had mild symptoms and no sign of pneumonia on chest imaging.

Moderate cases are those who had fever and respiratory symptoms and signs of pneumonia.

Severe cases were those who meet any of the falling criteria: 1) Shortness of breath, RR ≥30 times/min; 2) Oxygen saturation ≤ 93% at rest; 3) Alveolaroxygen partial pressure/fraction of inspiration O2 (PaO2/FiO2) ≤ 300 mmHg (1mmHg=0.133 kPa).

Critical cases are those who meet any of the following conditions: 1) respiratory failure requiring mechanical ventilation; 2) Shock; 3) Patients combined with other organ failure needed intensive care unit monitoring and treatment.

In our analysis, we combined severe and critical cases to a group to increase the statistical power.

Table S1. Extracted variables from included studies and corresponding descriptions for five severity measures.

Variables extracted	Description
author	First author of the study
city	Study location by city
province	Study location by province
date_start	The start date of the study
date_end	The end date of the study
event	Number of secondary cases with the presence of any of the severity measures
all	Number of all cases
design	Study design and setting, disaggregated into four categories as follows: outbreak investigations; mobile cabin hospital (i.e. Fangcang); admitted to emergency field hospitals and cross-sectional surveys
subvariant	Predominant subvariant during the study period (Omicron BA.1, BA.2, BA.5, BF.7, XBB or mixed subvariants)
ascertainment	Ascertainment method for COVID-19 cases
followup	Study follow up period or record period

age	Mean or median age of secondary cases (where unavailable, we derived such information using a weighted mean of all age groups of secondary cases)
vac	Proportion of vaccinated cases (primary doses or booster dose)
antiviral	Proportion of cases who received COVID-19 antiviral treatment
exposure	Whether the included cases had previous exposure to SARS-CoV-2
definition	The definition of the corresponding severity measure (if available)

Table S2. Summary of included studies of Omicron variant by characteristics.

Characteristic	Subgroup	Study	Number of studies
Providing information on cases with fever	NA	[2-59]	58
Providing information on cases with cough	NA	[2, 4, 6-16, 18-28, 30, 31, 33-55, 57-60]	53
Providing information on cases with sore throat	NA	[3, 4, 7, 9-13, 15, 16, 19-25, 27, 28, 30, 31, 33-44, 46-48, 50-55, 57, 59-61]	46
Providing information on symptomatic cases	NA	[2, 4-9, 12, 15-21, 25, 27-31, 34, 35, 37, 39, 41, 42, 44, 46, 47, 55, 57, 59-80]	54
Providing information on severe/critical cases	NA	[3-5, 7, 9-12, 17, 27, 28, 30, 33, 35, 43, 47, 48, 54-56, 59, 61, 62, 65, 67, 70, 73, 75-78, 81-87]	38

Providing information on deceased cases	NA	[3, 7, 8, 10, 11, 13, 17, 22, 29, 33, 41, 48, 54-56, 59, 61, 65, 73, 87]	20
Proportion of fully vaccinated cases (at least primary doses)	< 50%	[3, 10, 14, 15, 26, 29, 30, 43, 59, 81, 85]	11
	≥ 50%	[4-9, 11, 12, 16-18, 20-25, 27, 28, 31-38, 40, 41, 45-47, 51-54, 56-58, 60, 61, 67, 68, 70-72, 74-80, 82, 84, 86]	57
	NA	[2, 13, 19, 40, 42, 44, 48-50, 62-66, 69, 73, 83, 87]	18
Study design or setting	Outbreak investigation	[62, 63, 66-68, 72, 73, 77-80]	11
	Emergency hospital	[2-10, 12-17, 19-27, 29-33, 35, 36, 39-44, 47-49, 54, 56,	57

		57, 59, 61, 64, 65, 74-76, 81-87]	
	Fangcang hospital (mobile cabin/makeshift hospital)	[11, 18, 28, 34, 37, 55, 69, 71]	8
	Cross-sectional survey	[38, 45, 46, 50-53, 58, 60, 70]	10
Mean/median age, years	< 18	[4, 14, 26, 30, 59]	5
	18-59	[2-9, 11, 12, 16-18, 20-24, 27, 28, 31-42, 44-47, 50-58, 60, 61, 66-72, 74, 75, 77-80, 82, 84, 86, 87]	65
	≥ 60	[10, 13, 15, 29, 40, 43, 48, 49, 65, 81, 83, 85]	12
	NA	[62-64, 73]	4
Predominant subvariant	BA.1	[4, 8, 9, 17, 20, 22, 23, 27, 61, 63, 67, 82]	12

	BA.2	[2, 3, 5-7, 10-16, 18, 19, 21, 25, 26, 28-32, 34, 36, 37, 40, 41, 55, 59, 62, 64-66, 68, 69, 71, 73, 76, 77, 80, 81, 84]	42
	BA.5	[43, 46, 49, 51, 56, 57, 70, 72, 75, 85]	10
	BF.7	[33, 39, 47, 54, 58, 60]	6
	XBB	[38]	1
	Mixed subvariants	[35, 50, 52, 53, 74, 78, 79, 83]	8
	NA	[8, 24, 42, 44, 45, 48, 87]	7
Provided information on antiviral treatment among cases	Yes	[3, 6, 7, 10, 13, 16, 18, 22, 23, 33, 40, 41, 52, 59, 74, 76, 82, 86]	18
	No	[2, 4, 5, 8, 9, 11, 12, 14, 15, 17, 19-21, 24-32, 34-39, 42-	68

		51, 53-58, 60-73, 75, 77-81, 83-85, 87]	
Studies that conducted before relaxation of PHSMs on early December 2022	Yes	[2-37, 39-41, 47, 55, 59, 61-69, 71-82, 84, 86]	65
	No	[38, 42-46, 48-54, 56-58, 60, 70, 83, 85, 87]	21

Table S3. Number of computed estimates by each severity measure and characteristics.

	Fever	Cough	Sore throat	Symptomatic	Severe/critical	Death
Study setting						
Fangcang	6	6	5	6	1	1
Outbreak investigation	0	0	0	13	5	1
Emergency hospital	45	39	34	37	33	18
Cross-sectional survey	10	11	8	5	1	0
Predominant subvariant						
BA.1	8	7	7	8	7	4
BA.2	29	25	21	32	16	10
BA.5	8	7	4	7	5	0
BF.7	5	6	5	5	4	2

XBB	2	2	2	1	1	0
mixed	4	4	4	4	3	0
Age group						
< 18 yr	5	5	3	3	2	1
18-59 yr	48	43	38	49	29	13
≥ 60 yr	8	8	6	3	7	5
Vaccination coverage (at least primary doses)						
< 50%	9	8	6	4	7	5
≥ 50%	43	38	33	44	27	11
Antiviral treatment coverage						
>0%	14	12	10	5	7	9
0% or NA	47	44	37	56	33	11
Study period						
Prior to the relaxation	42	37	32	51	30	15

Post-relaxation	19	19	15	10	10	5

Table S4. Summary of findings of subgroup analysis and meta-regression of factors associated with severity of Omicron infections.

Variable	Number of computed estimates	Range, %	Median (IQR), %	*p*-value (moderator)	τ^2	Explained variance in heterogeneity
Percentage of cases with fever						
Overall	61	(7, 98)	45 (28, 65)		1.55	
Subvariant						
BA.1	8	(23, 43)	32 (24, 40)	< 0.01	1.18	19%
BA.2	29	(7, 98)	36 (19, 52)			
BA.5	8	(33, 98)	63 (52, 77)			
BF.7	5	(41, 73)	65 (45, 65)			
XBB	2	(54, 75)	65 (59, 70)			
Mixed	4	(28, 87)	68 (56, 74)			
Study setting						

Fangcang hospitals	6	(7, 61)	18 (8, 42)	< 0.01	1.21	22%
Emergency hospitals	45	(13, 98)	43 (28, 57)			
Cross-sectional surveys	10	(53, 98)	71 (65, 76)			
Age group						
< 18	5	(28, 98)	59 (56, 87)	0.11	1.51	3%
18-59	48	(7, 98)	43 (30, 65)			
≥ 60	8	(13, 69)	45 (20, 54)			
Primary vaccination coverage						
< 50%	11	(13, 98)	56 (32, 64)	0.16	1.52	2%
≥ 50%	43	(7, 98)	43 (28, 60)			

Antiviral uptake						
< 20%	16	(8, 87)	32 (24, 44)	0.91	1.09	0%
≥ 20%	7	(13, 73)	45 (19, 54)			
Study period						
Prior to the relaxation	42	(6, 98)	37 (23, 47)	< 0.01	1.08	30%
Post-relaxation	19	(33, 98)	65 (56, 76)			
Percentage of cases with cough						
Overall	56	(6, 92)	48 (36, 71)		1.04	
Subvariant						
BA.1	7	(29, 83)	45 (33, 56)	0.07	0.91	10%
BA.2	25	(6, 81)	41 (33, 59)			
BA.5	7	(31, 90)	73 (55, 79)			
BF.7	6	(25, 71)	47 (41, 71)			
XBB	2	(62, 74)	68 (65, 71)			

Mixed	4	(35, 92)	56 (41, 75)			
Study setting						
Fangcang hospitals	6	(6, 59)	40 (33, 44)	0.07	0.97	7%
Emergency hospitals	39	(12, 91)	49 (35, 71)			
Cross-sectional surveys	11	(31, 92)	62 (45, 72)			
Age group						
< 18	5	(17, 81)	37 (36, 63)	0.04	0.96	8%
18-59	43	(6, 92)	45 (34, 63)			
≥ 60	8	(29, 91)	72 (64, 79)			
Primary vaccination coverage						
< 50%	10	(29, 90)	67 (38, 72)	0.13	0.88	3%

≥ 50%	39	(6, 92)	43 (33, 62)			
Antiviral uptake						
< 20%	14	(24, 92)	37 (30, 59)	0.18	1.26	4%
≥ 20%	6	(25, 91)	72 (43, 78)			
Study period						
Prior to the relaxation	37	(6, 83)	41 (32, 58)	< 0.01	0.79	24%
Post-relaxation	19	(31, 92)	70 (55, 74)			
Percentage of cases with sore throat						
Overall	47	(0.5, 76)	30 (16, 42)		1.79	
Subvariant						
BA.1	7	(0.5, 43)	27 (23, 35)	< 0.01	1.07	31%
BA.2	21	(1, 72)	19 (14, 32)			
BA.5	4	(40, 76)	57 (51, 63)			
BF.7	5	(30, 60)	41 (38, 47)			

XBB	2	(69, 73)	71 (70, 72)			
Mixed	4	(33, 65)	53 (48, 57)			
Study setting						
Fangcang hospitals	5	(1, 38)	23 (14, 27)	< 0.01	1.34	25%
Emergency hospitals	34	(0.5, 72)	28 (16, 37)			
Cross-sectional surveys	8	(40, 76)	62 (54, 70)			
Age group						
< 18	3	(2, 30)	28 (15, 29)	0.27	1.79	0%
18-59	38	(0.5, 76)	32 (19, 46)			
≥ 60	6	(0.5, 72)	17 (11, 33)			
Primary vaccination coverage						

< 50%	8	(0.5, 72)	29 (18, 33)	0.43	1.64	0%
≥ 50%	34	(1, 76)	32 (19, 46)			
Antiviral uptake						
< 20%	11	(0.5, 65)	26 (17, 35)	0.92	1.44	0%
≥ 20%	6	(9, 38)	24 (17, 33)			
Study period						
Prior to the relaxation	32	(0.5, 72)	27 (15, 33)	0.02	1.61	10%
Post-relaxation	15	(0.5, 76)	54 (34, 62)			
Percentage of symptomatic cases						
Overall	61	(6, 100)	75 (53, 91)		3.38	
Subvariant						
BA.1	8	(60, 100)	94 (74, 99)	< 0.01	2.73	20%
BA.2	32	(7, 98)	55 (39, 82)			
BA.5	7	(6, 98)	84 (62, 87)			

BF.7	5	(62, 99)	88 (83, 91)			
XBB	1	(100, 100)	100 (100, 100)			
Mixed	4	(31, 85)	76 (60, 84)			
Study setting						
Fangcang hospitals	6	(10, 55)	20 (15, 24)	< 0.01	2.30	32%
Outbreak investigation	13	(6, 91)	53 (42, 70)			
Emergency hospitals	37	(31, 100)	85 (62, 97)			
Cross-sectional surveys	5	(63, 91)	84 (83, 87)			
Age group						
< 18	3	(55, 98)	82 (68, 90)	0.90	3.32	0%
18-59	49	(6, 100)	76 (55, 91)			
≥ 60	3	(39, 96)	85 (62, 85)			

Primary vaccination coverage						
< 50%	4	(39, 98)	84 (71, 89)	0.73	3.39	0%
≥ 50%	44	(6, 100)	74 (55, 90)			
Antiviral uptake						
< 20%	15	(20, 100)	83 (59, 98)	0.40	2.68	0%
≥ 20%	2	(43, 98)	70 (56, 84)			
Study period						
Prior to the relaxation	51	(6, 100)	66 (47, 87)	0.02	3.12	8%
Post-relaxation	10	(63, 100)	89 (85 ,97)			
Percentage of severe/critical cases						
Overall	40	(0, 63)	6 (0, 8)		5.88	
Subvariant						

BA.1	7	(0, 2)	0.2 (0, 0.8)	< 0.01	3.65	37%
BA.2	16	(0, 14)	0.2 (0, 1)			
BA.5	5	(0, 63)	46 (7, 46)			
BF.7	4	(0, 5)	2 (0.4, 3)			
XBB	1	(13, 13)	13 (13, 13)			
Mixed	3	(0, 42)	4 (2, 23)			
Study setting						
Fangcang hospitals	1	2 (2, 2)	2 (2, 2)	0.04	4.99	15%
Outbreak investigation	5	(0, 4)	0.2 (0.01, 0.7)			
Emergency hospitals	33	(0, 63)	0.7 (0, 11)			
Cross-sectional surveys	1	(46, 46)	46 (46, 46)			
Age group						

< 18	2	(0, 0.7)	0.4 (0.2, 0.6)	< 0.01	3.12	36%
18-59	29	(0, 46)	0.4 (0, 3)			
≥ 60	7	(4, 63)	33 (13, 44)			
Primary vaccination coverage						
< 50%	7	(0, 63)	4 (0.4, 29)	0.06	3.89	10%
≥ 50%	27	(0, 46)	0.4 (0, 3)			
Antiviral uptake						
< 20%	12	(0, 1.7)	0.05 (0, 0.4)	< 0.01	1.48	56%
≥ 20%	3	(0.7, 32.7)	4.8 (2.8, 18.7)			
Study period						
Prior to the relaxation	30	(0, 14)	0.2 (0, 1)	< 0.01	2.92	50%
Post-relaxation	10	(3, 63)	31 (13, 45)			

Percentage of death						
Overall	20	(0, 22)	0.001 (0, 2)		5.89	
Subvariant						
BA.1	4	(0, 0)	(0, 0)	0.42	4.66	0%
BA.2	10	(0, 2.4)	0.001 (0, 0.4)			
BF.7	2	(0, 2.7)	1.4 (0.7, 2)			
Study setting						
Fangcang hospitals	1	(0.004, 0.004)	(0.004, 0.004)	< 0.01	3.17	46%
Outbreak investigation	1	(0.003, 0.003)	(0.003, 0.003)			
Emergency hospitals	18	(0, 22)	0 (0, 2.4)			
Age group						
< 18	1	(0, 0)	0 (0, 0)	0.28	4.21	4%
18-59	13	(0, 5.7)	0 (0, 0.4)			

≥ 60	5	(0, 22)	2.3 (0.5, 2.4)			
Primary vaccination coverage						
< 50%	5	(0, 2.3)	0 (0, 0.5)	0.59	3.55	0%
≥ 50%	11	(0, 5.7)	0 (0, 0.2)			
Antiviral uptake						
< 20%	6	(0, 0)	0 (0, 0)	0.04	2.80	31%
≥ 20%	5	(0, 22)	0 (0, 0.5)			
Study period						
Prior to the relaxation	15	(0, 2)	0 (0, 0.2)	< 0.01	3.77	36%
Post-relaxation	5	(0, 22)	5 (3, 6)			

Table S5. Subgroup analysis and meta-regression of the language of articles associated with severity of Omicron infections.

Variable	Number of computed estimates	Median (IQR), %	τ^2	*p*-value (moderator)	Explained variance in heterogeneity
Percentage of cases with fever					
Language of the article					
English	50	46 (31, 65)	0.06	0.33	0.8%
Chinese	11	33 (25, 50)			
Percentage of cases with cough					
Language of the article					
English	48	50 (37, 71)	0.05	0.43	0.7%
Chinese	8	43 (32, 60)			
Percentage of cases with sore throat					
Language of the article					
English	40	32 (16, 48)	1.15	0.17	0.5%

Chinese	7	27 (12, 33)			
Percentage of symptomatic cases					
Language of the article					
English	48	83 (61, 92)	0.06	< 0.01	25%
Chinese	13	42 (19, 60)			
Percentage of severe/critical cases					
Language of the article					
English	32	0.7 (0, 8)	0.02	0.54	0%
Chinese	8	0.3 (0.004, 4)			
Percentage of deceased cases					
Language of the article					
English	13	0 (0, 5)	0.01	0.84	%
Chinese	7	0.4 (0.001, 4)			

Table S6. Sensitivity analysis with further accounting for vaccine coverage for association of epidemiological, demographic factors and public health measures with clinical severity profile for Omicron infection in China. Estimates are odds ratios and corresponding 95% confidence intervals.

	Percentage of symptomatic cases	Percentage of severe/critical cases	Percentage of death
Baseline model: adjusted for age group, study setting, vaccination coverage and subvariant			
Age group			
0-17	0.99 (0.71, 1.40)	0.99 (0.84, 1.17)	-
18-59	Reference		
≥ 60	0.81 (0.52, 1.24)	1.20 (1.03, 1.41) *	1.01 (0.99, 1.03)
Vaccination coverage	0.67 (0.34, 1.30)	0.89 (0.67, 1.19)	0.98 (0.96, 1.02)
Study setting			
Fangcang hospital	Reference		
Emergency hospital	1.57 (1.25, 1.98) *	0.96 (0.81, 1.13)	0.99 (0.98, 1.02)
Outbreak investigation	1.50 (1.14, 1.96) *	0.99 (0.82, 1.20)	-

Cross-sectional survey	1.77 (1.26, 2.49) *	1.30 (1.02, 1.66) *	-
Subvariant			
BA.1	Reference		
BA.2	0.77 (0.62, 0.95) *	0.95 (0.87, 1.04)	0.99 (0.98, 1.01)
BA.5	0.78 (0.60, 0.99) *	1.18 (1.05, 1.32) *	-
BF.7	0.91 (0.70, 1.19)	1.02 (0.92, 1.12)	1.00 (0.99, 1.02)
XBB	1.02 (0.64, 1.63)	1.11 (0.92, 1.34)	-
Mixed (any two or more of the above)	0.81 (0.62, 1.07)	1.01 (0.89, 1.15)	-
Baseline model with adjustment for government response index (GRI)			
GRI per 10-unit increase	0.92 (0.87, 0.98) *	0.97 (0.96, 0.99) *	1.00 (0.99, 1.01)
Baseline model with adjustment for containment and health index (CHI)			
CHI per 10-unit increase	0.93 (0.89, 0.98) *	0.98 (0.96, 0.99) *	1.00 (0.99, 1.01)
Baseline model with adjustment for stringency index (SI)			
SI per 10-unit increase	0.94 (0.89, 0.99) *	0.97 (0.96, 0.99) *	1.00 (0.99, 1.01)

Baseline model with adjustment for antiviral uptake			
Antiviral coverage #	1.19 (0.51, 2.82)	1.02 (0.98, 1.06)	0.98 (0.95, 1.00)
Baseline model with adjustment for study period			
Before relaxation (before December 8, 2022)	Reference		
After relaxation	1.61 (1.12, 2.32) *	1.21 (1.09, 1.35) *	-

The number of estimates for studies without missing information on factors in the baseline model and antiviral treatment coverage is 17, 14 and 10 for three clinical severity measures

* Statistically significant, $p < 0.05$

Table S7. Sensitivity analysis with further accounting for vaccine coverage for association of epidemiological, demographic factors and public health measures with symptom profile for Omicron infection in China. Estimates are odds ratios and corresponding 95% confidence intervals.

	Percentage of cases with fever	Percentage of cases with cough	Percentage of cases with sore throat
Baseline model: adjusted for age group, study setting and subvariant			
Age group			
0-17	1.15 (0.88, 1.52)	0.97 (0.71, 1.32)	0.98 (0.78, 1.23)
18-59	Reference		
≥ 60	0.79 (0.60, 1.05)	0.98 (0.71, 1.35)	1.08 (0.81, 1.43)
Vaccination coverage	0.67 (0.44, 1.02)	0.78 (0.48, 1.28)	1.02 (0.65, 1.59)
Study setting			
Fangcang hospital	Reference		
Emergency hospital	1.06 (0.89, 1.28)	1.01 (0.83, 1.24)	1.02 (0.86, 1.22)
Cross-sectional survey	1.39 (1.06, 1.83) *	0.91 (0.68, 1.21)	1.15 (0.89, 1.50)
Subvariant			

BA.1	Reference		
BA.2	0.97 (0.81, 1.16)	0.91 (0.75, 1.10)	0.98 (0.83, 1.16)
BA.5	1.22 (0.98, 1.52)	1.25 (0.99, 1.59)	1.31 (1.05, 1.63) *
BF.7	1.19 (0.96, 1.48)	1.05 (0.84, 1.32)	1.18 (0.97, 1.42)
XBB	1.11 (0.76, 1.61)	1.42 (0.97, 2.09)	1.41 (1.01, 1.97) *
Mixed (any two or more of the above)	1.16 (0.87, 1.54)	1.00 (0.73, 1.39)	1.14 (0.87, 1.49)
Baseline model with adjustment for government response index (GRI)			
GRI per 10-unit increase	0.99 (0.94, 1.04)	1.00 (0.95, 1.06)	1.01 (0.97, 1.06)
Baseline model with adjustment for containment and health index (CHI)			
CHI per 10-unit increase	0.99 (0.95, 1.04)	1.01 (0.96, 1.06)	1.01 (0.97, 1.06)
Baseline model with adjustment for stringency index (SI)			
SI per 10-unit increase	1.00 (0.96, 1.06)	1.02 (0.97, 1.07)	1.02 (0.98, 1.06)
Baseline model with adjustment for the antiviral uptake			
Antiviral coverage #	1.00 (0.63, 1.58)	1.21 (0.49, 2.92)	1.74 (1.19, 2.52) *

Baseline model with adjustment for the study period			
Before relaxation (before December 8, 2022)	Reference		
After relaxation	1.11 (0.80, 1.52)	1.31 (0.95, 1.81)	0.99 (0.96, 1.29)

[#] The number of estimates for studies without missing information on factors in the baseline model and antiviral treatment coverage is 20, 16 and 13 for three symptom measures

* Statistically significant, $p < 0.05$

Table S8. Sensitivity analysis of meta-regression by removing studies that judged to be at critical risk of bias.

	Percentage **of symptomatic cases**	Percentage **of severe/critical cases**	Percentage **of death**
Baseline model: adjusted for age group, study setting, vaccination coverage and subvariant			
Age group			
0-17	1.12 (0.86, 1.46)	1.04 (0.92, 1.18)	-
18-59	Reference		
≥ 60	1.03 (0.79, 1.34)	1.28 (1.18, 1.39) *	1.02 (0.98, 1.02)
Study setting			
Fangcang hospital	Reference		
Emergency hospital	1.54 (1.25, 1.88) *	0.96 (0.82, 1.14)	1.01 (1.00, 1.03) *
Outbreak investigation	1.40 (1.11, 1.77) *	0.97 (0.80, 1.17)	-
Cross-sectional survey	1.68 (1.23, 2.29) *	1.28 (1.01, 1.63) *	-
Subvariant			
BA.1	Reference		

BA.2	0.79 (0.66, 0.96) *	0.96 (0.89, 1.04)	1.00 (0.98, 1.01)
BA.5	0.79 (0.62, 1.01)	1.18 (1.06, 1.31) *	-
BF.7	0.92 (0.71, 1.19)	1.02 (0.92, 1.12)	1.01 (0.99, 1.03)
XBB	1.10 (0.71, 1.69)	1.13 (0.95, 1.36)	-
Mixed (any two or more of the above)	0.83 (0.64, 1.09)	1.06 (0.94, 1.19)	-
Baseline model with adjustment for government response index (GRI)			
GRI per 10-unit increase	0.93 (0.88, 0.99) *	0.97 (0.96, 0.99) *	1.00 (0.99, 1.01)
Baseline model with adjustment for containment and health index (CHI)			
CHI per 10-unit increase	0.94 (0.90, 0.99) *	0.97 (0.96, 0.99) *	1.00 (0.99, 1.01)
Baseline model with adjustment for stringency index (SI)			
SI per 10-unit increase	0.95 (0.91, 1.00)	0.97 (0.96, 0.99) *	1.00 (0.99, 1.01)
Baseline model with adjustment for antiviral uptake			
Antiviral coverage #	0.66 (0.21, 2.12)	1.03 (0.99, 1.08)	1.00 (0.89, 1.13)
Baseline model with adjustment for study period			

Before relaxation (before December 8, 2022)	Reference		
After relaxation	1.58 (1.10, 2.26) *	1.21 (1.11, 1.32) *	-
	Percentage **of cases with fever**	Percentage **of cases with cough**	Percentage **of cases with sore throat**
Baseline model: adjusted for age group, study setting and subvariant			
Age group			
0-17	1.28 (1.04, 1.56) *	1.03 (0.82, 1.30)	0.97 (0.80, 1.17)
18-59	Reference		
≥ 60	0.95 (0.81, 1.13)	1.16 (0.96, 1.39)	1.03 (0.87, 1.20)
Study setting			
Fangcang hospital	Reference		
Emergency hospital	1.08 (0.91, 1.28)	1.03 (0.84, 1.26)	1.03 (0.88, 1.21)
Cross-sectional survey	1.32 (1.02, 1.70) *	0.98 (0.74, 1.30)	1.18 (0.93, 1.50)

Subvariant			
BA.1	Reference		
BA.2	1.01 (0.86, 1.19)	0.95 (0.78, 1.14)	0.98 (0.85, 1.12)
BA.5	1.30 (1.06, 1.59) *	1.21 (0.96, 1.52)	1.29 (1.05, 1.58) *
BF.7	1.24 (1.01, 1.52) *	1.05 (0.84, 1.32)	1.16 (0.98, 1.38)
XBB	1.15 (0.81, 1.65)	1.33 (0.90, 1.96)	1.37 (1.01, 1.87) *
Mixed (any two or more of the above)	1.17 (0.90, 1.52)	1.21 (0.91, 1.61)	1.17 (0.93, 1.47)
Baseline model with adjustment for government response index (GRI)			
GRI per 10-unit increase	0.99 (0.94, 1.04)	0.99 (0.94, 1.05)	1.01 (0.97, 1.05)
Baseline model with adjustment for containment and health index (CHI)			
CHI per 10-unit increase	0.99 (0.95, 1.03)	0.99 (0.95, 1.04)	1.01 (0.97, 1.05)
Baseline model with adjustment for stringency index (SI)			
SI per 10-unit increase	1.01 (0.96, 1.05)	1.01 (0.96, 1.06)	1.02 (0.98, 1.06)
Baseline model with adjustment for the antiviral uptake			

Antiviral coverage #	0.69 (0.38, 1.28)	2.00 (0.83, 4.82)	1.85 (1.34, 2.55) *
Baseline model with adjustment for the study period			
Before relaxation (before December 8, 2022)	Reference		
After relaxation	1.17 (0.87, 1.57)	1.40 (1.02, 1.91) *	1.00 (0.79, 1.28)

SUPPLEMENTARY FIGURES

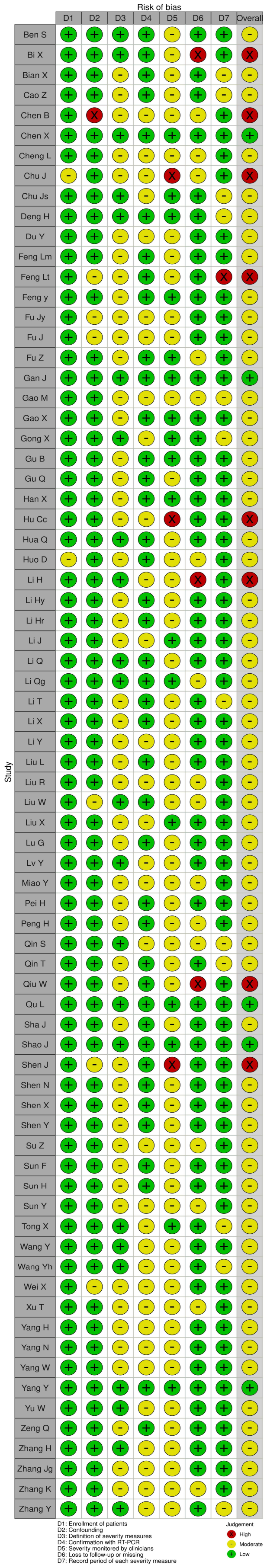

Figure S1. ROBINS-I assessment based on seven items including enrolment of all patients satisfying inclusion criteria (D1), information on confounders and efforts to address confounding (D2), clear definition of each severity measure (D3), case-ascertainment using RT-PCR (D4), severity measures evaluated by clinicians (D5), loss-to-follow < 5% (D6) and accurate period when the severity measure was assessed (D7). Each category was ranked as low (green), moderate (yellow) or high (red) risk of potential bias. The study ID (first author's name) is shown on the first column on the left of Figure S1. 12 Outbreak investigation studies were excluded for this assessment.

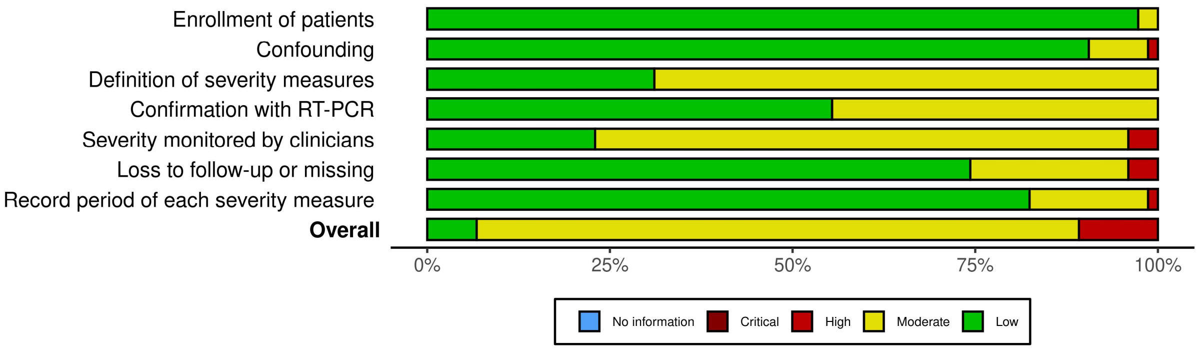

Figure S2. Overall ratings based on ROBINS-I assessment tool for 74 included studies (with 12 outbreak investigation studies excluded). Assessment of each included study is shown in Figure S1. A study with any item that is categorized as high risk will be determined to have an overall high risk of bias.

Study	Age	Vaccine coverage	Subvariant	Fever	Total	Proportion	95%−CI
**Emergency hospital**							
Ben S et al.	33	0.90	BA.2	100	321	0.31	[0.26; 0.37]
Bi X et al.	33		BA.2	34	65	0.52	[0.40; 0.65]
Bian X et al.	22	0.44	BA.2	263	380	0.69	[0.64; 0.74]
Cao Z et al.	40	0.93	BA.2	192	328	0.59	[0.53; 0.64]
Chen B et al.	44			576	602	0.96	[0.94; 0.97]
Cheng L et al.	75	0.35	BA.5	433	755	0.57	[0.54; 0.61]
Chu J et al.	41			196	327	0.60	[0.54; 0.65]
Feng Lm et al.	47	0.95	BA.1	17	40	0.42	[0.27; 0.59]
Feng Lt et al.	31	0.98	BA.1	40	92	0.43	[0.33; 0.54]
Fu J et al.	37	0.93		136	239	0.57	[0.50; 0.63]
Gao X et al.	33	0.93	BF.7	173	421	0.41	[0.36; 0.46]
Gu B et al.	34	0.83	BA.1	38	116	0.33	[0.24; 0.42]
Gu Q et al.	8	0.83	BA.2	25	89	0.28	[0.19; 0.39]
Han X et al.	45	0.84	BF.7	123	168	0.73	[0.66; 0.80]
Hu Cc et al.	2	0.00	BA.2	101	103	0.98	[0.93; 1.00]
Hua Q et al.	42	0.90	BA.2	96	225	0.43	[0.36; 0.49]
Li H et al.	75			117	223	0.52	[0.46; 0.59]
Li Hr et al.	36	0.83	BA.2	436	3265	0.13	[0.12; 0.15]
Li Hy et al.	46	0.79	BA.2	64	180	0.36	[0.29; 0.43]
Li Qg et al.	26	0.95	BA.1	96	384	0.25	[0.21; 0.30]
Li T et al.	36	0.89	.	132	430	0.31	[0.26; 0.35]
Li X et al.	6	0.29	BA.2	274	465	0.59	[0.54; 0.63]
Li Y et al.	6	0.41	BA.2	75	134	0.56	[0.47; 0.65]
Liu X et al.	43	0.94		33	119	0.28	[0.20; 0.37]
Lu G et al.	76	0.27	BA.2	273	1377	0.20	[0.18; 0.22]
Miao Y et al.	68		BA.5	145	210	0.69	[0.62; 0.75]
Peng H et al.	45	0.79	BA.2	69	508	0.14	[0.11; 0.17]
Qin T et al.	58	0.67		286	858	0.33	[0.30; 0.37]
Shao J et al.	52	0.56	BA.2	103	226	0.46	[0.39; 0.52]
Shen J et al.	72		BA.2	156	349	0.45	[0.39; 0.50]
Shen N et al.	3	0.14	BA.2	585	676	0.87	[0.84; 0.89]
Shen X et al.	71	0.40	BA.2	157	351	0.45	[0.39; 0.50]
Shen Y et al.	34	0.88	BA.2	17	136	0.12	[0.07; 0.19]
Sun F et al.	70	0.34	BA.2	130	1029	0.13	[0.11; 0.15]
Sun H et al.	48	0.85	BA.1	41	179	0.23	[0.17; 0.30]
Wang Y et al.	78		BA.2	144	760	0.19	[0.16; 0.22]
Wang Yh et al.	41	0.77	BF.7	2538	5594	0.45	[0.44; 0.47]
Wei X et al.	37	0.89	.	19	84	0.23	[0.14; 0.33]
Yang H et al.	59	0.72	BF.7	498	770	0.65	[0.61; 0.68]
Yang N et al.	32		BA.2	154	382	0.40	[0.35; 0.45]
Yang W et al.	35	0.87	BA.1	122	310	0.39	[0.34; 0.45]
Yu W et al.	41	0.83	BA.5	63	148	0.43	[0.34; 0.51]
Yu W et al.	41	0.88	BA.5	135	148	0.91	[0.85; 0.95]
Zhang H et al.	35	0.83	BA.2	698	2514	0.28	[0.26; 0.30]
Zhang Jg et al.	33	0.73	BA.2	51	169	0.30	[0.23; 0.38]
**Overall**				**10154**	**26249**		
**Prediction interval**							**[0.07; 0.90]**
Heterogeneity: I^2 = 98.7%							
**Cross−sectional survey**							
Du Y et al.	41	0.92		91	120	0.76	[0.67; 0.83]
Gao M et al.	42	0.89		69	124	0.56	[0.46; 0.65]
Huo D et al.	41	0.89	BF.7	206	317	0.65	[0.59; 0.70]
Huo D et al.	41	0.91	BA.5	65	90	0.72	[0.62; 0.81]
Qin S et al.	37			4390	6728	0.65	[0.64; 0.66]
Qu L et al.	32	1.00	BA.5	44	45	0.98	[0.88; 1.00]
Qu L et al.	29	1.00	XBB	7	13	0.54	[0.25; 0.81]
Su Z et al.	40	1.00		22737	32528	0.70	[0.69; 0.70]
Sun Y et al.	29	0.93	XBB	32320	42987	0.75	[0.75; 0.76]
Xu T et al.	37	0.96		10243	11718	0.87	[0.87; 0.88]
**Overall**				**70172**	**94670**		
**Prediction interval**							**[0.37; 0.93]**
Heterogeneity: I^2 = 99.5%							
**Fangcang hospital**							
Fu Jy et al.	34	1.00	BA.2	60	99	0.61	[0.50; 0.70]
Liu W et al.	42	0.75	BA.2	13611	208373	0.07	[0.06; 0.07]
Pei H et al.	45	0.81	BA.2	2027	25207	0.08	[0.08; 0.08]
Sha J et al.	39	0.87	BA.2	1742	3715	0.47	[0.45; 0.49]
Xu X et al.	46	0.74	BA.2	230	2818	0.08	[0.07; 0.09]
Zhang Y et al.	45	0.77	BA.2	34202	122151	0.28	[0.28; 0.28]
**Overall**				**51872**	**362363**		
**Prediction interval**							**[0.01; 0.88]**
Heterogeneity: I^2 = 100%							
Heterogeneity: I^2 = 99.9%							

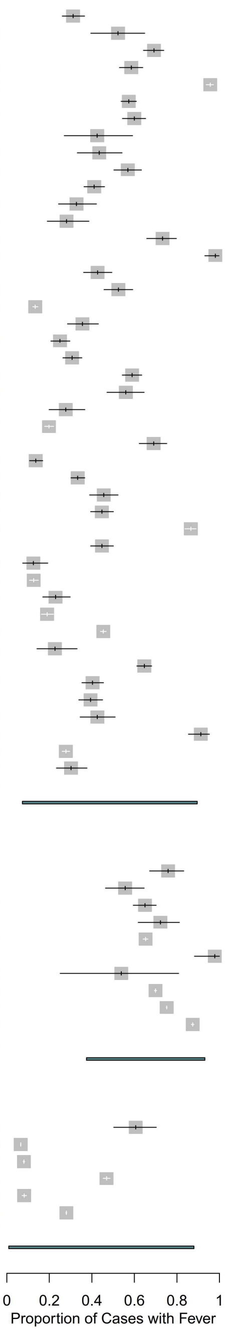

Figure S3. Estimates of the percentage of cases with fever stratified by study setting and their mean or median age (Age), primary and booster vaccination coverage (Vaccine coverage) and subvariant for Omicron cases in China. Prediction intervals were estimated based on Guddat et al.'s approach to provide a region where about 95% of the true effects are expected to be found [88]. Total: the number of all identified cases; Fever: number of cases presenting fever in identified cases.

Study	Age	Vaccine coverage	Subvariant	Cough	Total	Proportion	95%-CI
**Emergency hospital**							
Ben S et al.	33	0.90	BA.2	39	321	0.12	[0.09; 0.16]
Bi X et al.	33		BA.2	38	65	0.58	[0.46; 0.71]
Chen B et al.	44			422	602	0.70	[0.66; 0.74]
Cheng L et al.	75	0.35	BA.5	677	752	0.90	[0.88; 0.92]
Chu J et al.	41			186	327	0.57	[0.51; 0.62]
Feng Lm et al.	47	0.95	BA.1	33	40	0.82	[0.67; 0.93]
Feng Lt et al.	31	0.98	BA.1	41	92	0.45	[0.34; 0.55]
Fu J et al.	37	0.93		77	239	0.32	[0.26; 0.39]
Gao X et al.	33	0.93	BF.7	173	421	0.41	[0.36; 0.46]
Gu B et al.	34	0.83	BA.1	34	116	0.29	[0.21; 0.38]
Gu Q et al.	8	0.83	BA.2	15	89	0.17	[0.10; 0.26]
Han X et al.	45	0.84	BF.7	42	168	0.25	[0.19; 0.32]
Hu Cc et al.	2	0.00	BA.2	65	103	0.63	[0.53; 0.72]
Li H et al.	75			202	223	0.91	[0.86; 0.94]
Li Hr et al.	36	0.83	BA.2	770	3265	0.24	[0.22; 0.25]
Li Hy et al.	46	0.79	BA.2	67	180	0.37	[0.30; 0.45]
Li Qg et al.	26	0.95	BA.1	240	384	0.62	[0.57; 0.67]
Li T et al.	36	0.89	.	158	430	0.37	[0.32; 0.41]
Li X et al.	6	0.29	BA.2	171	465	0.37	[0.32; 0.41]
Li Y et al.	6	0.41	BA.2	108	134	0.81	[0.73; 0.87]
Liu X et al.	43	0.94		51	119	0.43	[0.34; 0.52]
Lu G et al.	76	0.27	BA.2	587	1377	0.43	[0.40; 0.45]
Miao Y et al.	68		BA.5	157	210	0.75	[0.68; 0.80]
Peng H et al.	45	0.79	BA.2	261	508	0.51	[0.47; 0.56]
Shao J et al.	52	0.56	BA.2	168	226	0.74	[0.68; 0.80]
Shen J et al.	72		BA.2	252	349	0.72	[0.67; 0.77]
Shen N et al.	3	0.14	BA.2	242	676	0.36	[0.32; 0.40]
Shen X et al.	71	0.40	BA.2	253	351	0.72	[0.67; 0.77]
Shen Y et al.	34	0.88	BA.2	46	136	0.34	[0.26; 0.42]
Sun H et al.	70	0.34	BA.1	302	1029	0.29	[0.27; 0.32]
Wang Y et al.	78		BA.2	542	760	0.71	[0.68; 0.75]
Wang Yh et al.	41	0.77	BF.7	3189	5594	0.57	[0.56; 0.58]
Yang H et al.	59	0.72	BF.7	548	770	0.71	[0.68; 0.74]
Yang N et al.	32		BA.2	157	382	0.41	[0.36; 0.46]
Yang W et al.	35	0.87	BA.1	151	310	0.49	[0.43; 0.54]
Yu W et al.	41	0.83	BA.5	93	148	0.63	[0.55; 0.71]
Yu W et al.	41	0.88	BA.5	124	148	0.84	[0.77; 0.89]
Zhang H et al.	35	0.83	BA.2	640	2514	0.25	[0.24; 0.27]
Zhang Jg et al.	33	0.73	BA.2	56	169	0.33	[0.26; 0.41]
**Overall**				**11377**	**24192**		
**Prediction interval**							**[0.13; 0.89]**
Heterogeneity: I^2 = 98.8%							
**Cross−sectional survey**							
Du Y et al.	41	0.92		85	120	0.71	[0.62; 0.79]
Gao M et al.	42	0.89		39	124	0.31	[0.23; 0.40]
Huo D et al.	41	0.89	BF.7	128	242	0.53	[0.46; 0.59]
Huo D et al.	41	0.91	BA.5	46	97	0.47	[0.37; 0.58]
Qin S et al.	37			4711	6728	0.70	[0.69; 0.71]
Qu L et al.	32	1.00	BA.5	33	45	0.73	[0.58; 0.85]
Qu L et al.	29	1.00	XBB	8	13	0.62	[0.32; 0.86]
Su Z et al.	40	1.00		11430	32528	0.35	[0.35; 0.36]
Sun Y et al.	29	0.93	XBB	31725	42987	0.74	[0.73; 0.74]
Xu T et al.	37			10741	11718	0.92	[0.91; 0.92]
Zhang K et al.	37	0.95		24362	57982	0.42	[0.42; 0.42]
**Overall**				**83308**	**152584**		
**Prediction interval**							**[0.16; 0.93]**
Heterogeneity: I^2 = 99.9%							
**Fangcang hospital**							
Fu Jy et al.	34	1.00	BA.2	58	99	0.59	[0.48; 0.68]
Liu W et al.	42	0.75	BA.2	12725	208373	0.06	[0.06; 0.06]
Pei H et al.	45	0.81	BA.2	11210	25207	0.44	[0.44; 0.45]
Sha J et al.	39	0.87	BA.2	1395	3715	0.38	[0.36; 0.39]
Xu X et al.	46	0.74	BA.2	880	2818	0.31	[0.30; 0.33]
Zhang Y et al.	45	0.77	BA.2	51181	122151	0.42	[0.42; 0.42]
**Overall**				**77449**	**362363**		
**Prediction interval**							**[0.03; 0.88]**
Heterogeneity: I^2 = 100%							
Heterogeneity: I^2 = 99.9%							

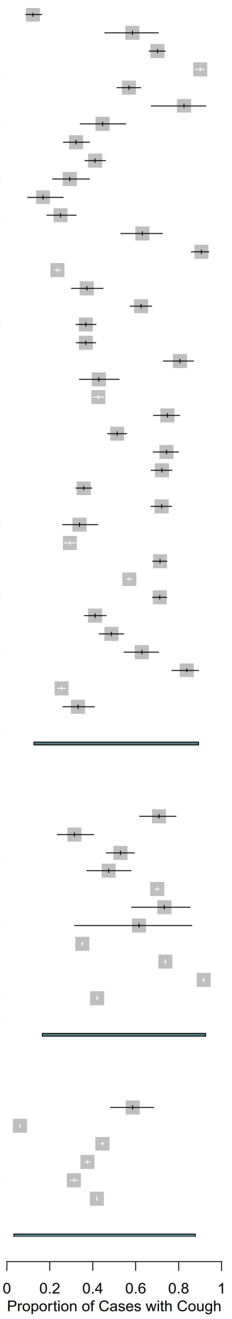

Figure S4. Estimates of the percentage of cases with cough stratified by study setting and their mean or median age (Age), primary and booster vaccination coverage (Vaccine coverage) and subvariant for Omicron cases in China. Prediction intervals were estimated based on Guddat et al.'s approach to provide a region where about 95% of the true effects are expected to be found [88]. Total: the number of all identified cases; Cough: number of cases presenting cough in identified cases.

Study	Age	Vaccine coverage	Subvariant	Sore throat	Total	Proportion	95%-CI
**Emergency hospital**							
Ben S et al.	33	0.90	BA.2	46	321	0.14	[0.11; 0.19]
Bian X et al.	22	0.44	BA.2	122	380	0.32	[0.27; 0.37]
Chen B et al.	44			3	602	0.00	[0.00; 0.01]
Chu J et al.	41			52	327	0.16	[0.12; 0.20]
Feng Lm et al.	47	0.95	BA.1	17	40	0.42	[0.27; 0.59]
Feng Lt et al.	31	0.98	BA.1	25	92	0.27	[0.18; 0.37]
Fu J et al.	37	0.93		53	239	0.22	[0.17; 0.28]
Gao X et al.	33	0.93	BF.7	173	421	0.41	[0.36; 0.46]
Gu B et al.	34	0.83	BA.1	37	116	0.32	[0.24; 0.41]
Gu Q et al.	8	0.83	BA.2	2	89	0.02	[0.00; 0.08]
Han X et al.	45	0.84	BF.7	51	168	0.30	[0.24; 0.38]
Li H et al.	75			19	223	0.09	[0.05; 0.13]
Li Hr et al.	36	0.83	BA.2	395	3265	0.12	[0.11; 0.13]
Li T et al.	36	0.89	.	82	430	0.19	[0.15; 0.23]
Li X et al.	6	0.29	BA.2	112	373	0.30	[0.25; 0.35]
Li Y et al.	6	0.41	BA.2	38	134	0.28	[0.21; 0.37]
Liu X et al.	43	0.94		39	119	0.33	[0.24; 0.42]
Lu G et al.	76	0.27	BA.2	247	1377	0.18	[0.16; 0.20]
Peng H et al.	45	0.79	BA.2	49	508	0.10	[0.07; 0.13]
Shao J et al.	52	0.56	BA.2	44	226	0.19	[0.15; 0.25]
Shen J et al.	72		BA.2	131	349	0.38	[0.32; 0.43]
Shen X et al.	71	0.40	BA.2	253	351	0.72	[0.67; 0.77]
Shen Y et al.	34	0.88	BA.2	46	136	0.34	[0.26; 0.42]
Sun H et al.	70	0.34	BA.1	5	1029	0.00	[0.00; 0.01]
Wang Y et al.	78		BA.2	125	760	0.16	[0.14; 0.19]
Wang Yh et al.	41	0.77	BF.7	2627	5594	0.47	[0.46; 0.48]
Yang H et al.	59	0.72	BF.7	290	770	0.38	[0.34; 0.41]
Yang N et al.	32		BA.2	122	382	0.32	[0.27; 0.37]
Yang W et al.	35	0.87	BA.1	118	310	0.38	[0.33; 0.44]
Yu W et al.	41	0.83	BA.5	168	296	0.57	[0.51; 0.62]
Yu W et al.	41	0.88	BA.5	87	148	0.59	[0.50; 0.67]
Zeng Q et al.	18	0.97	BA.1	100	380	0.26	[0.22; 0.31]
Zhang H et al.	35	0.83	BA.2	389	2514	0.15	[0.14; 0.17]
Zhang Jg et al.	33	0.73	BA.2	24	169	0.14	[0.09; 0.20]
**Overall**				**6091**	**22638**		
**Prediction interval**							**[0.02; 0.78]**
Heterogeneity: I^2 = 98.6%							
**Fangcang hospital**							
Fu Jy et al.	34	1.00	BA.2	38	99	0.38	[0.29; 0.49]
Liu W et al.	42	0.75	BA.2	2502	208373	0.01	[0.01; 0.01]
Pei H et al.	45	0.81	BA.2	5848	25207	0.23	[0.23; 0.24]
Sha J et al.	39	0.87	BA.2	512	3715	0.14	[0.13; 0.15]
Zhang Y et al.	45	0.77	BA.2	33103	122151	0.27	[0.27; 0.27]
**Overall**				**42003**	**359545**		
**Prediction interval**							**[0.00; 0.92]**
Heterogeneity: I^2 = 100%							
**Cross−sectional survey**							
Gao M et al.	42	0.89		50	124	0.40	[0.32; 0.50]
Qin S et al.	37			3534	6728	0.53	[0.51; 0.54]
Qu L et al.	32	1.00	BA.5	34	45	0.76	[0.60; 0.87]
Qu L et al.	29	1.00	XBB	9	13	0.69	[0.39; 0.91]
Su Z et al.	40	1.00		17659	32528	0.54	[0.54; 0.55]
Sun Y et al.	29	0.93	XBB	31181	42987	0.73	[0.72; 0.73]
Xu T et al.	37			7576	11718	0.65	[0.64; 0.66]
Zhang K et al.	37	0.95		34762	57982	0.60	[0.60; 0.60]
**Overall**				**94805**	**152125**		
**Prediction interval**							**[0.35; 0.81]**
Heterogeneity: I^2 = 99.8%							
Heterogeneity: I^2 = 99.9%							

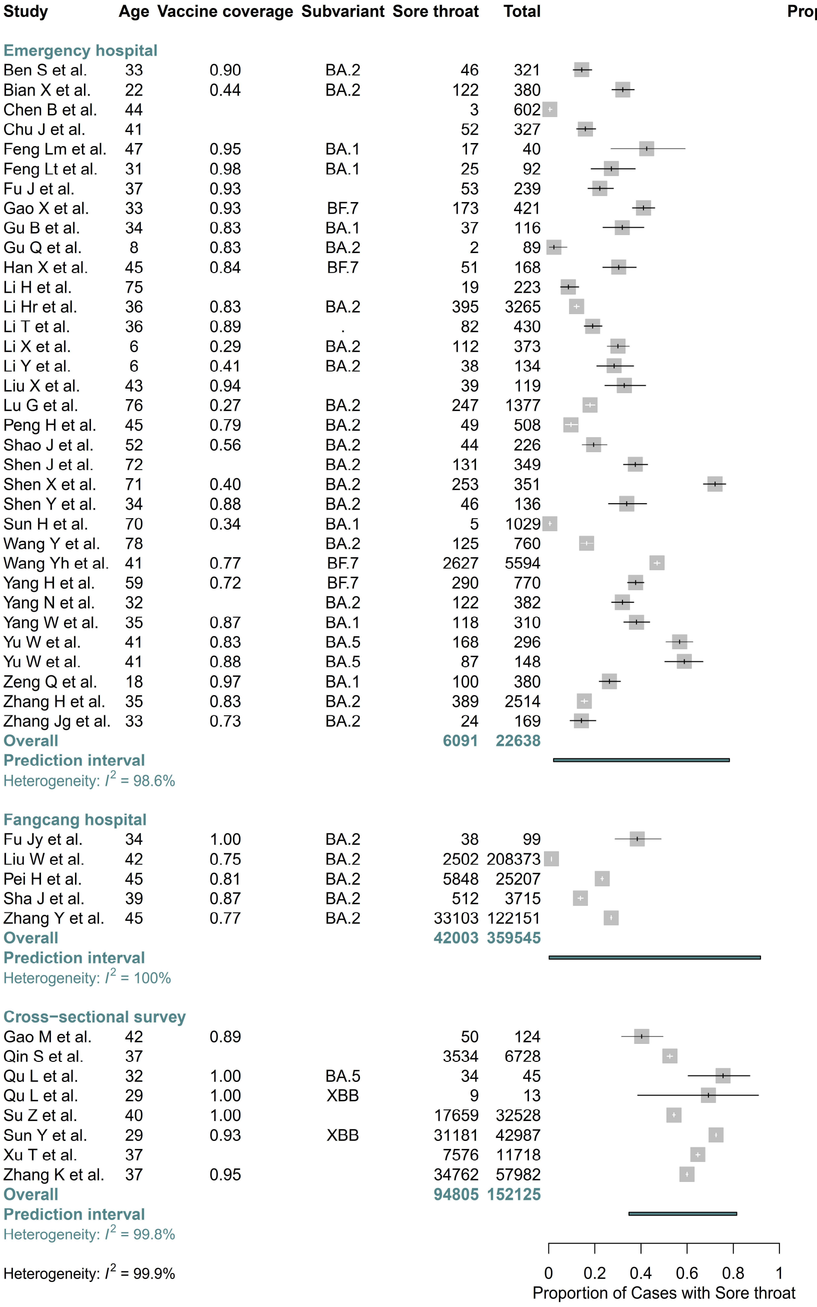

Figure S5. Estimates of the percentage of cases with sore throat stratified by study setting and their mean or median age (Age), primary and booster vaccination coverage (Vaccine coverage) and subvariant for Omicron cases in China. Prediction intervals were estimated based on Guddat et al.'s approach to provide a region where about 95% of the true effects are expected to be found [88]. Total: the number of all identified cases; Sore throat: number of cases presenting sore throat in identified cases.

Study	Age	Vaccine coverage	Subvariant	Symptomatic	Total	Proportion	95%−CI
**Emergency hospital**							
Ben S et al.	33	0.90	BA.2	213	321	0.66	[0.61; 0.72]
Bi X et al.	33		BA.2	61	65	0.94	[0.85; 0.98]
Chen B et al.	44			576	602	0.96	[0.94; 0.97]
Chu J et al.	41			317	327	0.97	[0.94; 0.99]
Deng H et al.	34	0.96	mixed	229	275	0.83	[0.78; 0.87]
Fu J et al.	37	0.93		214	239	0.90	[0.85; 0.93]
Gao X et al.	33	0.93	BF.7	369	421	0.88	[0.84; 0.91]
Gong X et al.	40	0.76	BA.5	33	54	0.61	[0.47; 0.74]
Gong X et al.	30	1.00	BF.7	24	39	0.62	[0.45; 0.77]
Gong X et al.	57	0.70	XBB	101	101	1.00	[0.96; 1.00]
Gu B et al.	34	0.83	BA.1	87	116	0.75	[0.66; 0.83]
Gu Q et al.	8	0.83	BA.2	49	89	0.55	[0.44; 0.66]
Hua Q et al.	42	0.90	BA.2	164	225	0.73	[0.67; 0.79]
Li Hr et al.	36	0.83	BA.2	1269	3265	0.39	[0.37; 0.41]
Li Hy et al.	46	0.79	BA.2	118	180	0.66	[0.58; 0.72]
Li J et al.	.		BA.2	145	154	0.94	[0.89; 0.97]
Li Q et al.	54	0.96		67	74	0.91	[0.81; 0.96]
Li T et al.	36	0.88	.	423	430	0.98	[0.97; 0.99]
Li X et al.	6	0.29	BA.2	380	465	0.82	[0.78; 0.85]
Li Y et al.	6	0.41	BA.2	131	134	0.98	[0.94; 1.00]
Liu X et al.	43	0.94		37	119	0.31	[0.23; 0.40]
Qiu W et al.	72		BA.2	1991	2347	0.85	[0.83; 0.86]
Shao J et al.	52	0.56	BA.2	222	226	0.98	[0.96; 1.00]
Shen X et al.	71	0.40	BA.2	301	351	0.86	[0.82; 0.89]
Shen Y et al.	34	0.88	BA.2	58	136	0.43	[0.34; 0.51]
Sun F et al.	70	0.34	BA.2	405	1029	0.39	[0.36; 0.42]
Sun H et al.	48	0.85	BA.1	178	179	0.99	[0.97; 1.00]
Wang Yh et al.	41	0.77	BF.7	7537	7562	1.00	[1.00; 1.00]
Wei X et al.	37	0.89	.	50	84	0.60	[0.48; 0.70]
Yang N et al.	32		BA.2	289	382	0.76	[0.71; 0.80]
Yang W et al.	35	0.87	BA.1	300	310	0.97	[0.94; 0.98]
Yang Y et al.	33	0.77	BA.2	1434	1721	0.83	[0.81; 0.85]
Yu W et al.	41	0.83	BA.5	130	148	0.88	[0.81; 0.93]
Yu W et al.	41	0.88	BA.5	145	148	0.98	[0.94; 1.00]
Zeng Q et al.	18	0.97	BA.1	380	380	1.00	[0.99; 1.00]
Zhang H et al.	35	0.83	BA.2	1454	2514	0.58	[0.56; 0.60]
Zhang Jg et al.	33	0.73	BA.2	90	169	0.53	[0.45; 0.61]
**Overall**				**19971**	**25381**		
**Prediction interval**							**[0.15; 1.00]**
Heterogeneity: $I^2 = 99\%$							
**Outbreak investigation**							
Fan X et al.	.		BA.2	13872	27036	0.51	[0.51; 0.52]
He W et al.	.		BA.1	123	175	0.70	[0.63; 0.77]
He W et al.	.		BA.2	138	1255	0.11	[0.09; 0.13]
He X et al.	35	0.84	BA.2	1069	1255	0.85	[0.83; 0.87]
Li D et al.	42	0.70	mixed	464	671	0.69	[0.66; 0.73]
Liu Y et al.	42	0.84	BA.2	1237	2334	0.53	[0.51; 0.55]
Wang K et al.	38	0.90	BA.5	69	1232	0.06	[0.04; 0.07]
Wang Z et al.	39		BA.2	672	1598	0.42	[0.40; 0.45]
Wu D et al.	26	1.00	BA.1	384	421	0.91	[0.88; 0.94]
Xian L et al.	.		BA.2	27644	397933	0.07	[0.07; 0.07]
Xian L et al.	.		BA.2	38744	74621	0.52	[0.52; 0.52]
Yin Z et al.	56	0.90	BA.2	99	151	0.66	[0.57; 0.73]
Zhao S et al.	56	0.92	mixed	51346	60534	0.85	[0.85; 0.85]
**Overall**				**135861**	**569216**		
**Prediction interval**							**[0.03; 0.97]**
Heterogeneity: $I^2 = 100\%$							
**Cross−sectional survey**							
Gao M et al.	42	0.89		78	124	0.63	[0.54; 0.71]
Huo D et al.	41	0.89	BF.7	202	242	0.83	[0.78; 0.88]
Huo D et al.	41	0.91	BA.5	84	97	0.87	[0.78; 0.93]
Liu R et al.	40	0.97		86384	102645	0.84	[0.84; 0.84]
Zhang K et al.	37	0.95		57982	63951	0.91	[0.90; 0.91]
**Overall**				**144730**	**167059**		
**Prediction interval**							**[0.47; 0.97]**
Heterogeneity: $I^2 = 99.7\%$							
**Fangcang hospital**							
Liu L et al.	43		BA.2	57	220	0.26	[0.20; 0.32]
Liu W et al.	42	0.75	BA.2	21249	208373	0.10	[0.10; 0.10]
Lv Y et al.	43	0.65	BA.2	2582	19156	0.13	[0.13; 0.14]
Sha J et al.	39	0.87	BA.2	2053	3715	0.55	[0.54; 0.57]
Xu X et al.	46	0.74	BA.2	562	2818	0.20	[0.18; 0.21]
Zhang Y et al.	45	0.77	BA.2	23453	122151	0.19	[0.19; 0.19]
**Overall**				**49956**	**356433**		
**Prediction interval**							**[0.03; 0.70]**
Heterogeneity: $I^2 = 99.9\%$							
Heterogeneity: $I^2 = 100.0\%$							

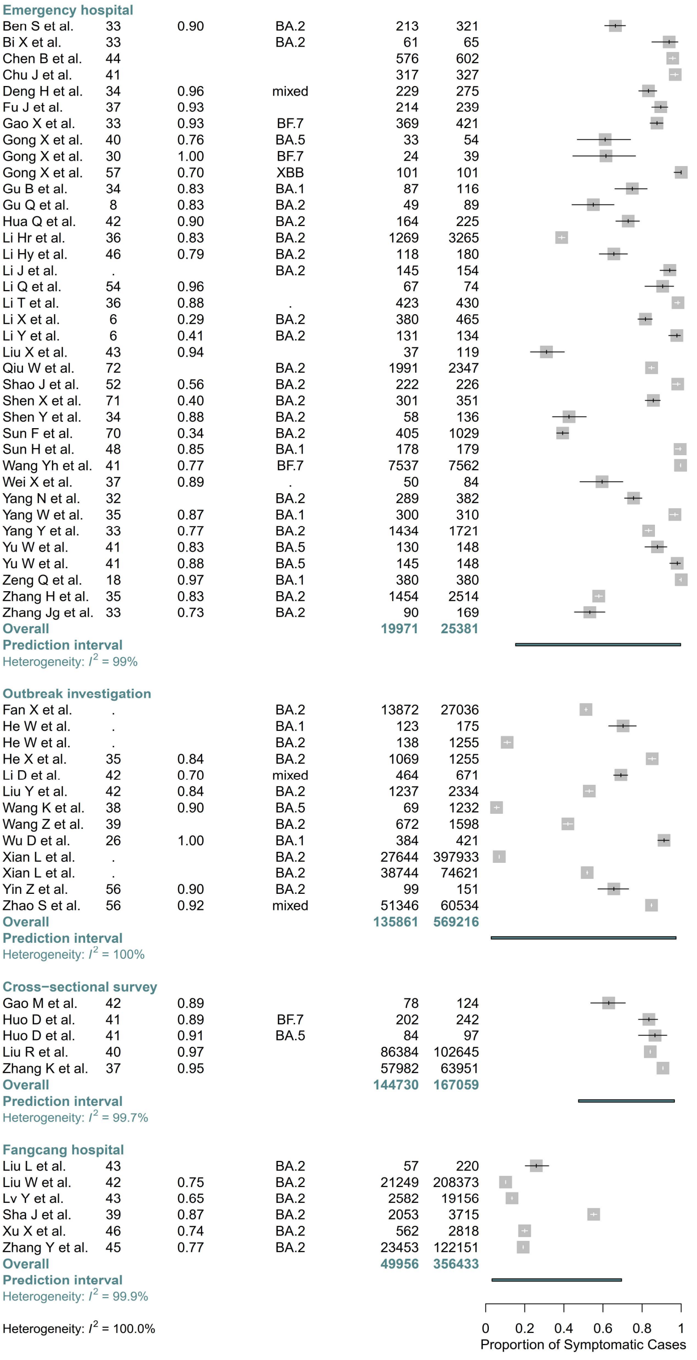

Figure S6. Estimates of the percentage of symptomatic cases stratified by study setting and their mean or median age (Age), primary and booster vaccination coverage (Vaccine coverage) and subvariant for Omicron cases in China. Prediction intervals were estimated based on Guddat et al.'s approach to provide a region where about 95% of the true effects are expected to be found [88]. Total: the number of all identified cases; Symptomatic: number of cases with symptomatic infection in identified cases.

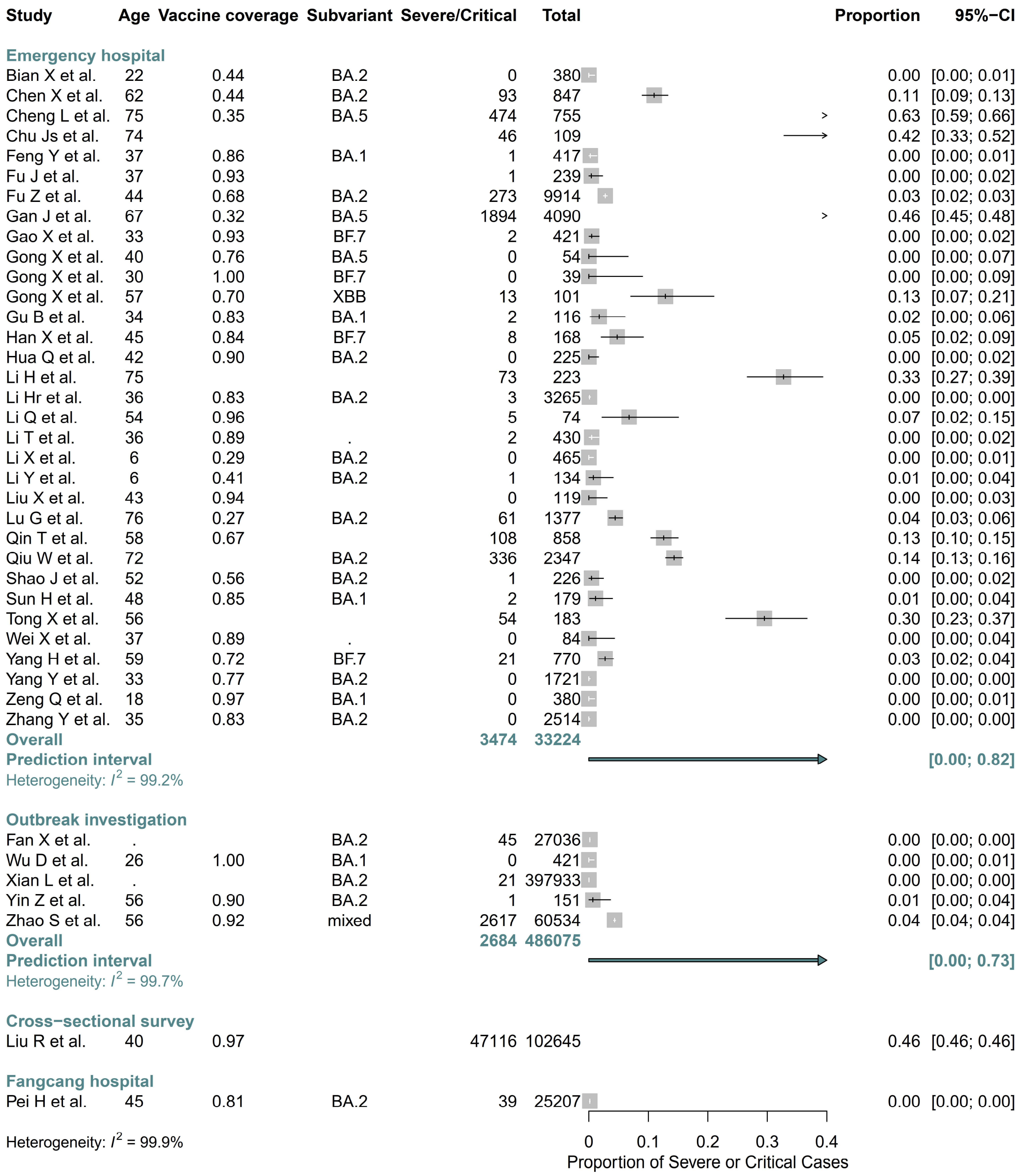

Figure S7. Estimates of the percentage of severe/critical cases stratified by study setting and their mean or median age (Age), primary and booster vaccination coverage (Vaccine coverage) and subvariant for Omicron cases in China. Prediction intervals were estimated based on Guddat et al.'s approach to provide a region where about 95% of the true effects are expected to be found [88]. Total: the number of all identified cases; Severe/Critical: number of cases with severe/critical illness in identified cases.

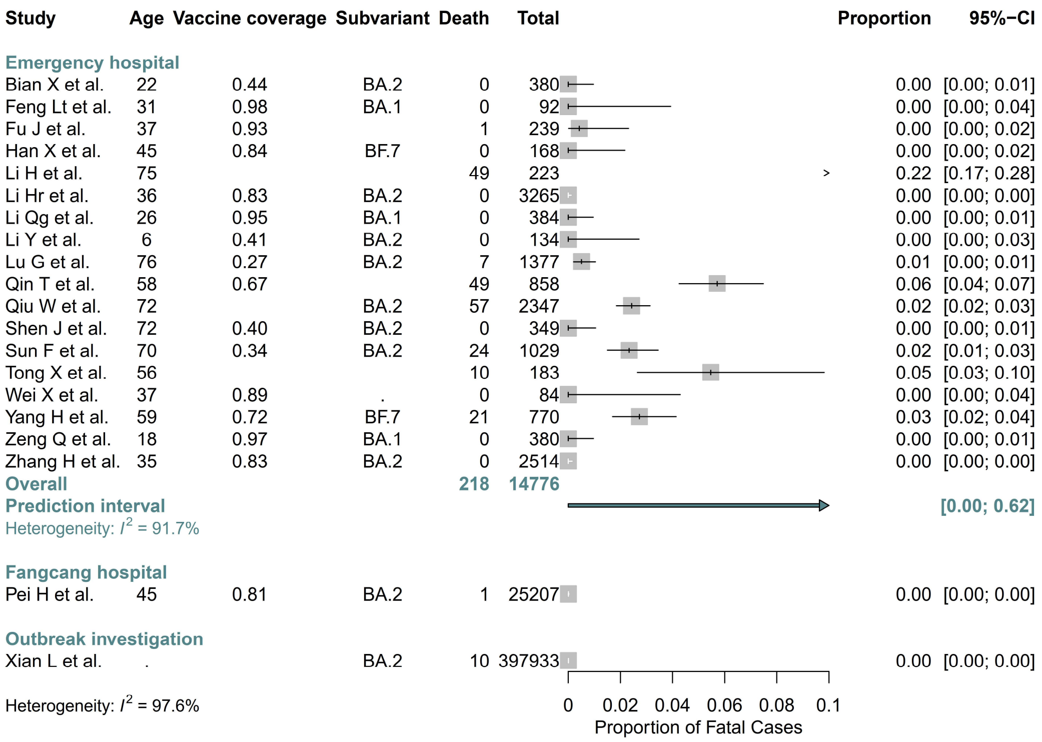

Figure S8. Estimates of the percentage of deaths stratified by study setting and their mean or median age (Age), primary and booster vaccination coverage (Vaccine coverage) and subvariant for Omicron cases in China. Prediction intervals were estimated based on Guddat et al.'s approach to provide a region where about 95% of the true effects are expected to be found [88]. Total: the number of all identified cases; Death: number of fatal cases in identified cases.

References

1. Wu Y, Feng X, Gong M, Han J, Jiao Y, Li S, et al. Evolution and major changes of the diagnosis and treatment protocol for COVID-19 patients in China 2020-2023. Health Care Sci. 2023;2(3):135-52. Epub 2023/05/17. doi: 10.1002/hcs2.45. PubMed PMID: 38939112; PubMed Central PMCID: PMCPMC11080729.
2. Bi X, Zhang Y, Pan J, Chen C, Zheng Y, Wang J, et al. Differences Between Omicron Infections and Fever Outpatients: Comparison of Clinical Manifestations and Initial Routine Hematology Indicators. Infect Drug Resist. 2022;15:5111-20. Epub 2022/09/08. doi: 10.2147/idr.S378990. PubMed PMID: 36068832; PubMed Central PMCID: PMCPMC9441180.
3. Bian XL, Guo Z, Zhang K, Li MC, Wu ZM, Jiang Q, et al. [Clinical features of children and their family members with family clusters of SARS-CoV-2 Omicron variant infection in Shanghai, China: an analysis of 380 cases]. Zhongguo Dang Dai Er Ke Za Zhi. 2022;24(10):1085-91. Epub 2022/10/29. doi: 10.7499/j.issn.1008-8830.2205179. PubMed PMID: 36305107; PubMed Central PMCID: PMCPMC9628002.
4. Gu B, Yao L, Zhu XY, Zou T, Feng YJ, Yan JY, et al. Comparison of initial clinic characteristics of hospitalized patients in Suzhou City during the COVID-19 Omicron wave with ancestral variant wave. Ther Adv Respir Dis. 2022;16:17534666221110346. Epub 2022/07/08. doi: 10.1177/17534666221110346. PubMed PMID: 35796535; PubMed Central PMCID: PMCPMC9340419.
5. Hua Q, Zheng D, Yu B, Tan X, Chen Q, Wang L, et al. Effectiveness of Inactivated COVID-19 Vaccines against COVID-19 Caused by the SARS-CoV-2 Delta and Omicron Variants: A Retrospective Cohort Study. Vaccines (Basel). 2022;10(10). Epub 2022/10/28. doi: 10.3390/vaccines10101753. PubMed PMID: 36298618; PubMed Central PMCID: PMCPMC9611172.
6. Li H, Zhu M, Zhang P, Yan X, Niu J, Wang Z, et al. Milder symptoms and shorter course in patients with re-positive COVID-19: A cohort of 180 patients from Northeast China. Front Microbiol. 2022;13:989879. Epub 2022/10/29. doi: 10.3389/fmicb.2022.989879. PubMed PMID: 36304945; PubMed Central PMCID: PMCPMC9593080.
7. Li H, Zhu X, Yu R, Qian X, Huang Y, Chen X, et al. The effects of vaccination on the disease severity and factors for viral clearance and hospitalization in Omicron-infected patients: A retrospective observational cohort study from recent regional outbreaks in China. Front Cell Infect Microbiol. 2022;12:988694. Epub 2022/11/25. doi: 10.3389/fcimb.2022.988694. PubMed PMID: 36420118; PubMed Central PMCID: PMCPMC9677104.
8. Li Q, Liu X, Li L, Hu X, Cui G, Sun R, et al. Comparison of clinical characteristics between SARS-CoV-2 Omicron variant and Delta variant infections in China. Front Med (Lausanne). 2022;9:944909. Epub 2022/11/01. doi: 10.3389/fmed.2022.944909. PubMed PMID: 36313994; PubMed Central PMCID: PMCPMC9597612.
9. Li T, Han M, Wang J, Zhou C, Mu H. Clinical characteristics and risks of the convalescent COVID-19 patients with re-detectable positive RNA test: a 430 patients with Omicron infected cross-sectional survey in Tianjin, China. J Infect Public Health. 2022;15(12):1409-14. Epub 2022/11/18. doi: 10.1016/j.jiph.2022.11.011. PubMed PMID: 36395665; PubMed Central PMCID: PMCPMC9651934.

10. Lu G, Zhang Y, Zhang H, Ai J, He L, Yuan X, et al. Geriatric risk and protective factors for serious COVID-19 outcomes among older adults in Shanghai Omicron wave. Emerg Microbes Infect. 2022;11(1):2045-54. Epub 2022/08/05. doi: 10.1080/22221751.2022.2109517. PubMed PMID: 35924388; PubMed Central PMCID: PMCPMC9448390.
11. Ying-Hao P, Yuan-Yuan G, Hai-Dong Z, Qiu-Hua C, Xue-Ran G, Hai-Qi Z, et al. Clinical characteristics and analysis of risk factors for disease progression of patients with SARS-CoV-2 Omicron variant infection: A retrospective study of 25207 cases in a Fangcang hospital. Front Cell Infect Microbiol. 2022;12:1009894. Epub 2022/11/18. doi: 10.3389/fcimb.2022.1009894. PubMed PMID: 36389157; PubMed Central PMCID: PMCPMC9659624.
12. Shao J, Fan R, Hu J, Zhang T, Lee C, Huang X, et al. Clinical Progression and Outcome of Hospitalized Patients Infected with SARS-CoV-2 Omicron Variant in Shanghai, China. Vaccines (Basel). 2022;10(9). Epub 2022/09/24. doi: 10.3390/vaccines10091409. PubMed PMID: 36146487; PubMed Central PMCID: PMCPMC9503563.
13. Shen J, Wu L, Wang P, Shen X, Jiang Y, Liu J, et al. Clinical characteristics and short-term recovery of hyposmia in hospitalized non-severe COVID-19 patients with Omicron variant in Shanghai, China. Front Med (Lausanne). 2022;9:1038938. Epub 2022/11/25. doi: 10.3389/fmed.2022.1038938. PubMed PMID: 36419783; PubMed Central PMCID: PMCPMC9676490.
14. Shen N, Wu YF, Chen YW, Fang XY, Zhou M, Wang WY, et al. Clinical characteristics of pediatric cases infected with the SARS-CoV-2 Omicron variant in a tertiary children's medical center in Shanghai, China. World J Pediatr. 2023;19(1):87-95. Epub 2022/10/18. doi: 10.1007/s12519-022-00621-6. PubMed PMID: 36251118; PubMed Central PMCID: PMCPMC9574794.
15. Shen X, Wang P, Shen J, Jiang Y, Wu L, Nie X, et al. Neurological Manifestations of hospitalized patients with mild to moderate infection with SARS-CoV-2 Omicron variant in Shanghai, China. J Infect Public Health. 2023;16(2):155-62. Epub 2022/12/20. doi: 10.1016/j.jiph.2022.12.005. PubMed PMID: 36535135; PubMed Central PMCID: PMCPMC9726211.
16. Shen Y, Ai J, Lin N, Zhang H, Li Y, Wang H, et al. An open, prospective cohort study of VV116 in Chinese participants infected with SARS-CoV-2 omicron variants. Emerg Microbes Infect. 2022;11(1):1518-23. Epub 2022/05/18. doi: 10.1080/22221751.2022.2078230. PubMed PMID: 35579892; PubMed Central PMCID: PMCPMC9176639.
17. Wei X, Fang Z, Zhu H, Gan C, Huang M. [Myocardium damage and electrocardiogram characteristics of patients infected with SARS-CoV-2 Omicron variant in Zhuhai]. Chinese Journal of Arteriosclerosis. 2022;30(10):884-9. doi: 10.20039/j.cnki.1007-3949.2022.10.008.
18. Xu X, Zhou S, Chen C, Li J, Wu H, Jin G, et al. Efficacy and safety of Reyanning mixture in patients infected with SARS-CoV-2 Omicron variant: A prospective, open-label, randomized controlled trial. Phytomedicine. 2023;108:154514. Epub 2022/11/06. doi: 10.1016/j.phymed.2022.154514. PubMed PMID: 36334390; PubMed Central PMCID: PMCPMC9616513.
19. Yang N, Wang C, Huang J, Dong J, Ye J, Fu Y, et al. Clinical and Pulmonary CT Characteristics of Patients Infected With the SARS-CoV-2 Omicron Variant Compared With Those of Patients Infected With the Alpha Viral Strain. Front Public Health.

2022;10:931480. Epub 2022/07/30. doi: 10.3389/fpubh.2022.931480. PubMed PMID: 35903393; PubMed Central PMCID: PMCPMC9315283.
20. Yang W, Yang S, Wang L, Zhou Y, Xin Y, Li H, et al. Clinical characteristics of 310 SARS-CoV-2 Omicron variant patients and comparison with Delta and Beta variant patients in China. Virol Sin. 2022;37(5):704-15. Epub 2022/08/11. doi: 10.1016/j.virs.2022.07.014. PubMed PMID: 35948265; PubMed Central PMCID: PMCPMC9357284.
21. Zhang J, Chen N, Zhao D, Zhang J, Hu Z, Tao Z. Clinical Characteristics of COVID-19 Patients Infected by the Omicron Variant of SARS-CoV-2. Front Med (Lausanne). 2022;9:912367. Epub 2022/05/27. doi: 10.3389/fmed.2022.912367. PubMed PMID: 35615088; PubMed Central PMCID: PMCPMC9125333.
22. Feng L, Liu X, Zhang L. [Clinical observation of Xuanfei Baidu Granule in the treatment of COVID-19 (Omicron)]. Tianjin Journal of Traditional Chinese Medicine. 2022;39(05):545-50.
23. Feng L, Wang X, Li L, Wu Q. [Comparison in clinical characteristics of native and imported people infected with the SARS-CoV-2 Omicron variant]. Shandong Medical Journal. 2022;62(21):30-3.
24. Fu J, Mao X, Jiang L. [Relevant Investigation and Physical Analysis of Patients with Persistently Positive of COVID-19 Omicron Variant]. Journal of Emergency in Traditional Chinese Medicine. 2022;31(10):1800-3.
25. Gu Q, Cao Z, Zhang Y. [Clinical analysis of 89 children infected with SARS-CoV-2 in Lianyungang]. Journal of Xuzhou Medical University. 2022;42(07):543-6.
26. Hu C, Xu P, Xu L, Zhang Y, Zhou J, Wang L, et al. [Short-term persistent symptoms in preschool children with mild/common coronavirus disease 2019 caused by Omicron variant infection after discharge:a follow-up study]. Chinese Journal of Contemporary Pediatrics. 2022;24(12):1289-94.
27. Sun H, Zhang Y, Shi C, Liu X, Zhao G, Zhao Q, et al. [Factor analysis of nucleic acid turning negative time of novel coronavirus Omicron infection patients treated by integrated traditional Chinese and Western medicine in Tianjin area]. Tianjin Journal of Traditional Chinese Medicine. 2022;39(05):551-5.
28. Y. Z, Xie X, Yu X, Liang H, Liu K, Li L, et al. [Epidemiological characteristics of SARS-CoV-2 Omicron variant patients in a Shanghai Fangcang hospital]. Infectious Disease Information. 2022;35(04):311-5+41.
29. Sun F, Zhang Y, Li Y, Zhang H, Liu Q, Ai J, et al. [Inactivated vaccine protects against severe outcome among adults aged over 60 years during SARS-CoV-2 Omicron variant predominance:evidence from a single-center cohort]. Journal of Microbes and Infections. 2022;17(04):209-19.
30. Li X, Wu L, Qu Y, Cao M, Feng J, Huang H, et al. Clinical characteristics and vaccine effectiveness against SARS-CoV-2 Omicron subvariant BA.2 in the children. Signal Transduct Target Ther. 2022;7(1):203. Epub 2022/06/29. doi: 10.1038/s41392-022-01023-w. PubMed PMID: 35764610; PubMed Central PMCID: PMCPMC9240082.
31. Ben S, Gao F, Xu Z, Zhang R, Zhang X, Wang N, et al. The role of hematological parameters in asymptomatic and non-severe cases of Omicron variant infection. Virol J. 2024;21(1):143. Epub 2024/06/25. doi: 10.1186/s12985-024-02414-x. PubMed PMID: 38915037; PubMed Central PMCID: PMCPMC11197332.
32. Cao Z, Sun F, Ding H, Tian Z, Cui Y, Yang W, et al. A retrospective analysis of the influencing factors of nucleic acid CT value fluctuation in COVID-19 patients infected with

Omicron variant virus in Changchun city. Front Public Health. 2024;12:1377135. Epub 2024/07/01. doi: 10.3389/fpubh.2024.1377135. PubMed PMID: 38947348; PubMed Central PMCID: PMCPMC11211536.
33. Han X, Chen J, Chen L, Jia X, Fan Y, Zheng Y, et al. Comparative Analysis of Clinical and CT Findings in Patients with SARS-CoV-2 Original Strain, Delta and Omicron Variants. Biomedicines. 2023;11(3). Epub 2023/03/30. doi: 10.3390/biomedicines11030901. PubMed PMID: 36979880; PubMed Central PMCID: PMCPMC10046064.
34. Liu W, Gong F, Zheng X, Pei L, Wang X, Yang S, et al. Factors associated with prolonged viral shedding of SARS-CoV-2 Omicron variant infection in Shanghai: A multicenter, retrospective, observational study. J Med Virol. 2023;95(12):e29342. Epub 2023/12/22. doi: 10.1002/jmv.29342. PubMed PMID: 38130170.
35. Liu X, Zhang P, Chen M, Zhou H, Yue T, Xu M, et al. Epidemiological and clinical features of COVID-19 inpatients in Changsha, China: A retrospective study from 2020 to 2022. Heliyon. 2023;9(12):e22873. Epub 2023/12/21. doi: 10.1016/j.heliyon.2023.e22873. PubMed PMID: 38125480; PubMed Central PMCID: PMCPMC10731055.
36. Peng H, Xiang T, Xu F, Jiang Y, Zhong L, Peng Y, et al. Redistribution and Activation of CD16brightCD56dim NK Cell Subset to Fight against Omicron Subvariant BA.2 after COVID-19 Vaccination. Microorganisms. 2023;11(4). Epub 2023/04/28. doi: 10.3390/microorganisms11040940. PubMed PMID: 37110363; PubMed Central PMCID: PMCPMC10145754.
37. Sha J, Meng C, Sun J, Sun L, Gu R, Liu J, et al. Clinical and upper airway characteristics of 3715 patients with the Omicron variant of SARS-Cov-2 in Changchun, China. J Infect Public Health. 2023;16(3):422-9. Epub 2023/02/03. doi: 10.1016/j.jiph.2023.01.013. PubMed PMID: 36731245; PubMed Central PMCID: PMCPMC9854235.
38. Sun Y, Duan Y, Qian J, Qu Y, Wang Y, Fan G, et al. A Large-Scale Online Survey on Clinical Severity and Associated Risk Factors for SARS-CoV-2 Omicron Infection - China, April-May 2023. China CDC Wkly. 2024;6(15):305-11. Epub 2024/05/13. doi: 10.46234/ccdcw2024.059. PubMed PMID: 38736994; PubMed Central PMCID: PMCPMC11082054.
39. Wang Y, Yu G, Shi J, Zhang X, Huo J, Li M, et al. Retrospective study about clinical severity and epidemiological analysis of the COVID-19 Omicron subvariant lineage-infected patients in Hohhot, China. BMC Infect Dis. 2024;24(1):206. Epub 2024/02/16. doi: 10.1186/s12879-024-09084-8. PubMed PMID: 38360539; PubMed Central PMCID: PMCPMC10870667.
40. Wang Y, Zhao D, Chen X, Liu X, Xiao W, Feng L. The effect of nirmatrelvir-ritonavir on viral clearance and length of hospital stay in patients infected with SARS-CoV-2 omicron variants. Influenza Other Respir Viruses. 2023;17(2):e13095. Epub 2023/02/28. doi: 10.1111/irv.13095. PubMed PMID: 36843224; PubMed Central PMCID: PMCPMC9946694.
41. Zhang H, Weng Z, Zheng Y, Zheng M, Chen W, He H, et al. Epidemiological and clinical features of SARS-CoV-2 Omicron variant infection in Quanzhou, Fujian province: a retrospective study. Sci Rep. 2023;13(1):22152. Epub 2023/12/14. doi: 10.1038/s41598-023-49098-x. PubMed PMID: 38092887; PubMed Central PMCID: PMCPMC10719353.
42. Chen B, Shi J, Chen J, Qiu Y. Clinical characteristics of current COVID-19 rehabilitation outpatients in China. Open Med (Wars). 2023;18(1):20230771. Epub

2023/09/11. doi: 10.1515/med-2023-0771. PubMed PMID: 37693834; PubMed Central PMCID: PMCPMC10487391.
43. Cheng Ll, Li Zt, Wu Hk, Li F, Qiu Y, Wang T, et al. Clinical and pathogen features of COVID-19-associated infections during an Omicron strain outbreak in Guangzhou, China. Microbiol Spectr. 2024;12(10):e0340623. Epub 2024/09/06. doi: 10.1128/spectrum.03406-23. PubMed PMID: 39240085; PubMed Central PMCID: PMCPMC11448415.
44. Chu J, Dai Q, Dong C, Kong X, Tian H, Li C, et al. The serological IgG and neutralizing antibody of SARS-CoV-2 omicron variant reinfection in Jiangsu Province, China. Front Public Health. 2024;12:1364048. Epub 2024/06/14. doi: 10.3389/fpubh.2024.1364048. PubMed PMID: 38873290; PubMed Central PMCID: PMCPMC11169644.
45. Du Y, Li C, Zhao W, Li J, Zhao L, Guo H, et al. Multimodal neuroimaging exploration of the mechanisms of sleep quality deterioration after SARS-CoV-2 Omicron infection. BMC Med. 2024;22(1):271. Epub 2024/06/27. doi: 10.1186/s12916-024-03487-9. PubMed PMID: 38926881; PubMed Central PMCID: PMCPMC11210028.
46. Gao M, Xing X, Hao W, Zhang X, Zhong K, Lu C, et al. Diverse immune responses in vaccinated individuals with and without symptoms after omicron exposure during the recent outbreak in Guangzhou, China. Heliyon. 2024;10(2):e24030. Epub 2024/01/31. doi: 10.1016/j.heliyon.2024.e24030. PubMed PMID: 38293451; PubMed Central PMCID: PMCPMC10827461.
47. Gao X, Wang F, Liu H, Chai J, Tian G, Yao L, et al. BF.7: a new Omicron subvariant characterized by rapid transmission. Clin Microbiol Infect. 2024;30(1):137-41. Epub 2023/10/07. doi: 10.1016/j.cmi.2023.09.018. PubMed PMID: 37802303.
48. Li H, Jia X, Wang Y, Lv Y, Wang J, Zhai Y, et al. Differences in the severity and mortality risk factors for patients hospitalized for COVID-19 pneumonia between the early wave and the very late stage of the pandemic. Front Med (Lausanne). 2023;10:1238713. Epub 2023/10/16. doi: 10.3389/fmed.2023.1238713. PubMed PMID: 37841011; PubMed Central PMCID: PMCPMC10568453.
49. Miao Y, Ren Y, Ren T. Clinical Characteristics Profile of COVID-19 Patients with Omicron Variant Admitted in a Tertiary Hospital, Central China. Int J Gen Med. 2023;16:2365-71. Epub 2023/06/16. doi: 10.2147/ijgm.S409478. PubMed PMID: 37325696; PubMed Central PMCID: PMCPMC10263011.
50. Qin S, Li Y, Wang L, Zhao X, Ma X, Gao GF. Assessment of vaccinations and breakthrough infections after adjustment of the dynamic zero-COVID-19 strategy in China: an online survey. Emerg Microbes Infect. 2023;12(2):2258232. Epub 2023/09/11. doi: 10.1080/22221751.2023.2258232. PubMed PMID: 37691586; PubMed Central PMCID: PMCPMC10512888.
51. Qu L, Xie C, Qiu M, Yi L, Liu Z, Zou L, et al. Characterizing Infections in Two Epidemic Waves of SARS-CoV-2 Omicron Variants: A Cohort Study in Guangzhou, China. Viruses. 2024;16(4). Epub 2024/04/27. doi: 10.3390/v16040649. PubMed PMID: 38675989; PubMed Central PMCID: PMCPMC11053513.
52. Xu T, Chen Y, Zhan W, Chung KF, Qiu Z, Huang K, et al. Profiles of Cough and Associated Risk Factors in Nonhospitalized Individuals With SARS-CoV-2 Omicron Variant Infection: Cross-Sectional Online Survey in China. JMIR Public Health Surveill. 2024;10:e47453. Epub 2024/02/05. doi: 10.2196/47453. PubMed PMID: 38315527; PubMed Central PMCID: PMCPMC10877488.

53. Su Z, Li Y, Xie Y, Huang Z, Cheng A, Zhou X, et al. Acute and long COVID-19 symptoms and associated factors in the omicron-dominant period: a nationwide survey via the online platform Wenjuanxing in China. BMC Public Health. 2024;24(1):2086. Epub 2024/08/02. doi: 10.1186/s12889-024-19510-w. PubMed PMID: 39090598; PubMed Central PMCID: PMCPMC11295386.
54. Yang H, Wang Z, Zhang Y, Xu M, Wang Y, Zhang Y, et al. Clinical characteristics and factors for serious outcomes among outpatients infected with the Omicron subvariant BF.7. J Med Virol. 2023;95(8):e28977. Epub 2023/08/28. doi: 10.1002/jmv.28977. PubMed PMID: 37635385.
55. Fu J, Cui K, Li Z, Xie Z, Tian H, Liao Y, et al. [Epidemiological characteristics analysis of
the SARS-CoV-2 Omicron variant infection in Gannan area]. Infectious Disease Information. 2023;36(05):440-4.
56. Qin T, Zheng X, Feng J, Lu W, Zhou K, Ling Y, et al. [Risk Factors and Their Influence Analysis on Mortality of 858 COVID-19 Pneumonia Patients with Omicron Variant]. GuangXi Sciences. 2023;30(02):369-74.
57. Yu W, Guo Y, Hu T, Liu Y, Fan Q, Guo L, et al. Incidence and severity of SARS-CoV-2 reinfection, a multicenter cohort study in Shanghai, China. J Med Virol. 2023;95(8):e28997. Epub 2023/08/04. doi: 10.1002/jmv.28997. PubMed PMID: 37537950.
58. Huo D, Yu T, Shen Y, Pan Y, Li F, Cui S, et al. A Comparison of Clinical Characteristics of Infections with SARS-CoV-2 Omicron Subvariants BF.7.14 and BA.5.2.48 - China, October-December 2022. China CDC Wkly. 2023;5(23):511-5. Epub 2023/07/05. doi: 10.46234/ccdcw2023.096. PubMed PMID: 37404291; PubMed Central PMCID: PMCPMC10316608.
59. Li YC, Ma Z, Zhong HY, You HL. Clinical characteristics of children with omicron SARS-CoV-2 infection in Changchun, China from march to april 2022: A retrospective study. Front Pediatr. 2022;10:990944. Epub 2022/12/03. doi: 10.3389/fped.2022.990944. PubMed PMID: 36458144; PubMed Central PMCID: PMCPMC9705729.
60. Zhang K, Zhong X, Fan X, Yu D, Chen Z, Zhao C, et al. Asymptomatic infection and disappearance of clinical symptoms of COVID-19 infectors in China 2022-2023: a cross-sectional study. Sci Rep. 2024;14(1):18232. Epub 2024/08/07. doi: 10.1038/s41598-024-68162-8. PubMed PMID: 39107338; PubMed Central PMCID: PMCPMC11303783.
61. Zeng QL, Lv YJ, Liu XJ, Jiang ZY, Huang S, Li WZ, et al. Clinical Characteristics of Omicron SARS-CoV-2 Variant Infection After Non-mRNA-Based Vaccination in China. Front Microbiol. 2022;13:901826. Epub 2022/07/19. doi: 10.3389/fmicb.2022.901826. PubMed PMID: 35847120; PubMed Central PMCID: PMCPMC9279136.
62. Fan X, Lu S, Bai L, Liu H, Fu J, Jin X, et al. Preliminary Study of the Protectiveness of Vaccination Against the COVID-19 in the Outbreak of VOC Omicron BA.2 - Jilin City, Jilin Province, China, March 3-April 12, 2022. China CDC Wkly. 2022;4(18):377-80. Epub 2022/06/11. doi: 10.46234/ccdcw2022.081. PubMed PMID: 35686205; PubMed Central PMCID: PMCPMC9167613.
63. He W, Yu F, Wei Y, Sun W, Ren D, Wu Z, et al. [Epidemiology of infections with SARS-CoV-2 Omicron variant in Jiangsu Province,China]. Journal of Nanjing Medical University (Natural Sciences). 2022;42(11):1614-20.
64. Li J, Song R, Yuan Z, Xu Z, Suo L, Wang Q, et al. Protective Effect of Inactivated COVID-19 Vaccines against Progression of SARS-CoV-2 Omicron and Delta Variant

Infections to Pneumonia in Beijing, China, in 2022. Vaccines (Basel). 2022;10(8). Epub 2022/08/27. doi: 10.3390/vaccines10081215. PubMed PMID: 36016103; PubMed Central PMCID: PMCPMC9413898.
65. Qiu W, Shi Q, Chen F, Wu Q, Yu X, Xiong L. The derived neutrophil to lymphocyte ratio can be the predictor of prognosis for COVID-19 Omicron BA.2 infected patients. Front Immunol. 2022;13:1065345. Epub 2022/11/22. doi: 10.3389/fimmu.2022.1065345. PubMed PMID: 36405724; PubMed Central PMCID: PMCPMC9666892.
66. Wang Z, Liu B, Qi X, Zhang R, Bian S, Jiang M. [Epidemiological characteristics of local COVID-19 cases in Zhejiang Province]. Preventive Medicine. 2022;34(12):1240-4. doi: 10.19485/j.cnki.issn2096-5087.2022.12.010.
67. Wu D, Ye Y, Tang L, Wang AB, Zhang R, Qian ZH, et al. A case-case study on the effect of primary and booster immunization with China-produced COVID-19 vaccines on prevention of pneumonia and viral load among vaccinated persons infected by Delta and Omicron variants. Emerg Microbes Infect. 2022;11(1):1950-8. Epub 2022/07/20. doi: 10.1080/22221751.2022.2103455. PubMed PMID: 35850623; PubMed Central PMCID: PMCPMC9359169.
68. He X, Liao Y, Liang Y, Yu J, Gao W, Wan J, et al. Transmission characteristics and inactivated vaccine effectiveness against transmission of the SARS-CoV-2 Omicron BA.2 variant in Shenzhen, China. Front Immunol. 2023;14:1290279. Epub 2024/01/23. doi: 10.3389/fimmu.2023.1290279. PubMed PMID: 38259438; PubMed Central PMCID: PMCPMC10800792.
69. Liu L, Zhang J, Zhu H, Pan B, Ma S, Li M, et al. [Distribution of Traditional Chinese Medicine Constitution Types and Prevention and Control Strategies in Patients Infected with the Omicron Variant of Severe Acute Respiratory Syndrome Coronavirus 2 in Shanghai, China:An Analysis of 220 Cases]. Journal of Anhui University of Chinese Medicine. 2022;41(05):1-5.
70. Liu R, Zhang Y, Ma J, Wang H, Lan Y, Tang X. Epidemiological features of SARS-CoV-2 Omicron infection under new control strategy: a cross-sectional study of the outbreak since December 2022 in Sichuan, China. BMC Public Health. 2023;23(1):2463. Epub 2023/12/09. doi: 10.1186/s12889-023-17361-5. PubMed PMID: 38066518; PubMed Central PMCID: PMCPMC10709916.
71. Lv Y, Liu D, Li J. [Influencing factors of nucleic acid negative conversion in patients with asymptomatic and mild COVID-19 induced by the Omicron variant of SARS-COV-2]. Shaanxi Medical Journal. 2022;51(12):1511-5.
72. Wang K, Guo Z, Zeng T, Sun S, Lu Y, Wang J, et al. Transmission Characteristics and Inactivated Vaccine Effectiveness Against Transmission of SARS-CoV-2 Omicron BA.5 Variants in Urumqi, China. JAMA Netw Open. 2023;6(3):e235755. Epub 2023/03/31. doi: 10.1001/jamanetworkopen.2023.5755. PubMed PMID: 36995713; PubMed Central PMCID: PMCPMC10064257.
73. Xian L, Lin J, Yu S, Zhao Y, Zhao P, Cao G. [Epidemiological characteristics of SARS-CoV-2 infection outbreak in Shanghai in the Spring of 2022]. Shanghai Journal of Preventive Medicine. 2022;34(04):294-9. doi: 10.19428/j.cnki.sjpm.2022.22058.
74. Deng H, Mai Y, Liu H, Guan J. Clinical characteristics of liver injury in SARS-CoV-2 Omicron variant- and Omicron subvariant-infected patients. Ann Hepatol. 2023;28(1):100763. Epub 2022/10/02. doi: 10.1016/j.aohep.2022.100763. PubMed PMID: 36182032; PubMed Central PMCID: PMCPMC9515007.

75. Gong X, Peng L, Wang F, Liu J, Tang Y, Peng Y, et al. Repeated Omicron infection dampens immune imprinting from previous vaccination and induces broad neutralizing antibodies against Omicron sub-variants. J Infect. 2024;89(2):106208. Epub 2024/06/23. doi: 10.1016/j.jinf.2024.106208. PubMed PMID: 38908522.
76. Yang Y, Guo L, Yuan J, Xu Z, Gu Y, Zhang J, et al. Viral and antibody dynamics of acute infection with SARS-CoV-2 omicron variant (B.1.1.529): a prospective cohort study from Shenzhen, China. Lancet Microbe. 2023;4(8):e632-e41. Epub 2023/07/18. doi: 10.1016/s2666-5247(23)00139-8. PubMed PMID: 37459867.
77. Yin Z, Fang Q, Wen T, Zheng C, Fu C, Wang S, et al. Effectiveness of COVID-19 vaccines against SARS-CoV-2 Omicron variants during two outbreaks from March to May 2022 in Quzhou, China. Hum Vaccin Immunother. 2023;19(1):2163813. Epub 2023/01/28. doi: 10.1080/21645515.2022.2163813. PubMed PMID: 36704960; PubMed Central PMCID: PMCPMC10012893.
78. Zhao S, Luo K, Guo Y, Fang M, Sun Q, Dai Z, et al. Analysis of Factors Influencing the Clinical Severity of Omicron and Delta Variants. Trop Med Infect Dis. 2023;8(6). Epub 2023/06/27. doi: 10.3390/tropicalmed8060330. PubMed PMID: 37368748; PubMed Central PMCID: PMCPMC10301725.
79. Liu D, Feng S, Sha F, Liao Y, Xie X, Huang F, et al. Inactivated SARS-CoV-2 Vaccine Booster Against Omicron Infection Among Quarantined Close Contacts. JAMA Netw Open. 2023;6(10):e2339507. Epub 2023/10/25. doi: 10.1001/jamanetworkopen.2023.39507. PubMed PMID: 37878315; PubMed Central PMCID: PMCPMC10600580.
80. Liu Y, Chai YH, Wu YF, Zhang YW, Wang L, Yang L, et al. Risk factors associated with indoor transmission during home quarantine of COVID-19 patients. Front Public Health. 2023;11:1170085. Epub 2023/05/30. doi: 10.3389/fpubh.2023.1170085. PubMed PMID: 37250088; PubMed Central PMCID: PMCPMC10213781.
81. Chen X, Wang H, Ai J, Shen L, Lin K, Yuan G, et al. Identification of CKD, bedridden history and cancer as higher-risk comorbidities and their impact on prognosis of hospitalized Omicron patients: a multi-centre cohort study. Emerg Microbes Infect. 2022;11(1):2501-9. Epub 2022/09/16. doi: 10.1080/22221751.2022.2122581. PubMed PMID: 36106526; PubMed Central PMCID: PMCPMC9621241.
82. Feng Y, Shao H, Gong X, Song Z, Xie Y, Qi S, et al. 'Dynamic zero-COVID' policy and viral clearance during an omicron wave in Tianjin, China: a city-wide retrospective observational study. BMJ Open. 2022;12(12):e066359. Epub 2022/12/16. doi: 10.1136/bmjopen-2022-066359. PubMed PMID: 36521897; PubMed Central PMCID: PMCPMC9755905.
83. Chu J, Hua L, Liu X, Xiong H, Jiang F, Zhou W, et al. Superoxide dismutase alterations in COVID-19: implications for disease severity and mortality prediction in the context of omicron variant infection. Front Immunol. 2024;15:1362102. Epub 2024/03/11. doi: 10.3389/fimmu.2024.1362102. PubMed PMID: 38464514; PubMed Central PMCID: PMCPMC10921560.
84. Fu Z, Liang D, Zhang W, Shi D, Ma Y, Wei D, et al. Host protection against Omicron BA.2.2 sublineages by prior vaccination in spring 2022 COVID-19 outbreak in Shanghai. Front Med. 2023;17(3):562-75. Epub 2023/03/24. doi: 10.1007/s11684-022-0977-3. PubMed PMID: 36949347; PubMed Central PMCID: PMCPMC10033297.
85. Gan J, Zhang H, Wu J, Liu Y, Liu P, Cheng R, et al. Effect of inactivated vaccine boosters against severe and critical COVID-19 during the Omicron BA.5 wave: A

retrospective analysis of hospitalized patients in China. J Med Virol. 2024;96(2):e29402. Epub 2024/02/21. doi: 10.1002/jmv.29402. PubMed PMID: 38380744.
86. Li Q, Wang Y, Liu H, Peng H, Xiang J, Guo S. Imaging Progression Under Low Respiratory Viral Load of SARS-CoV-2 Omicron Variant Infection: A Retrospective Study in China. Infect Drug Resist. 2023;16:6795-806. Epub 2023/10/31. doi: 10.2147/idr.S417062. PubMed PMID: 37904829; PubMed Central PMCID: PMCPMC10613413.
87. Tong X, Yang Y, Hu W. [Immune response patterns of patients with novel coronavirus pneumonia]. Zhejiang Clinical Medicine. 2023;25(09):1340-2.
88. Guddat C, Grouven U, Bender R, Skipka G. A note on the graphical presentation of prediction intervals in random-effects meta-analyses. Syst Rev. 2012;1:34. Epub 2012/07/31. doi: 10.1186/2046-4053-1-34. PubMed PMID: 22839660; PubMed Central PMCID: PMCPMC3552946.
